# The process of student engagement in school health promotion: a scoping review

**DOI:** 10.1186/s12889-025-22121-8

**Published:** 2025-03-19

**Authors:** Julia C. Kontak, Caitlyn Macrae, Hilary A. T. Caldwell, Becky Feicht, Stephanie Heath, Camille L. Hancock Friesen, Sara F. L. Kirk

**Affiliations:** 1 Faculty of Health, Dalhousie University, 5968 College Street, PO Box 15000, Halifax, NS B3H 4R2 Canada; 2 Healthy Populations Institute, Dalhousie University, 1318 Robie Street, Halifax, Nova Scota B3H 3E2 Canada; 3 School of Health and Human Performance, Dalhousie University, PO Box 15000, Halifax, NS B3H 4R2 Canada; 4 Research Power Inc, 114 Ochterloney St, Dartmouth, NS B2Y 1C7 Canada; 5 Division of Pediatric Cardiothoracic Surgery, Children’s Nebraska, University of Nebraska Medical Center, 8200 Dodge Street, Omaha, NE 68114 USA

**Keywords:** Health promotion, School health, Youth engagement, Participatory

## Abstract

**Background:**

Health Promoting Schools (HPS) is a whole school model that strengthens and maintains a healthy school environment. While a key component of HPS is the engagement of students, there is little known about the strategies for, facilitators of, and barriers to, student engagement. The purpose of this scoping review was to summarize and characterize the evidence on the process of student engagement in school health promotion, with a focus on whole school models like HPS.

**Methods:**

This scoping review followed the Joanna Briggs Institute guidelines and the Arksey and O’Malley scoping review framework. The Preferred Reporting Items for Systematic Reviews and Meta-Analyses extension for scoping reviews guided reporting. Eligibility included sources examining the process of student engagement in school health promotion for children and youth (ages 5–19) in any country, who attended a private or public school. Both published and unpublished sources were included. Databases searched were: CINAHL, ERIC, MEDLINE, Scopus, and Google Scholar. Relevant organisational websites and sources identified by experts were also reviewed. Two independent reviewers screened the title, abstract, and full text of the sources. Descriptive analysis was conducted for quantitative data, and content analysis was employed for qualitative data.

**Results:**

1740 sources were screened, 133 citations were eligible for full text review and a total of 50 sources were included: 38 peer-reviewed publications, 7 grey literature sources, 2 peer-reviewed publications from reference-checking and 3 sources recommended by experts. The majority of articles reported on primary research (*n* = 34), employed qualitative methods (*n* = 28) and over half of all sources were published from European institutions/organizations (*n* = 26). Process strategies for student engagement predominantly related to participatory mechanisms including reflection and visioning, determining priorities and action-oriented learning. A wide range of intersecting facilitators and barriers were identified, with school structures largely acting as a barrier and adult approaches to engagement being a facilitator.

**Conclusion:**

This scoping review described the strategies, facilitators and barriers involving the process of student engagement in school health promotion. The results can inform the development and implementation of future student engagement strategies to strengthen school health promotion actions.

**Supplementary Information:**

The online version contains supplementary material available at 10.1186/s12889-025-22121-8.

## Introduction

Schools have long been identified as an optimal setting for health promotion [1–3]. As most children and youth spend almost half of their waking hours within a school environment, it is an ideal place for students to form and adopt health behaviours during a time of transition and development. Health Promoting Schools (HPS), also referred to as Comprehensive School Health [4], is a whole school approach that aims to create the conditions necessary to help mitigate precursors to disease, as well as enhance and support student competencies related to the various facets of health and well-being. The World Health Organization (WHO) describes HPS as “a school that constantly strengthens its capacity as a safe and healthy setting for teaching, learning, and working” [5, p. 8]. In practice, HPS approaches vary based on context, yet collectively focus on similar components outlined by the International Union for Health Promotion and Education (IUHPE), including school policies, the social and physical environment, the school curriculum, and family and community partnerships [6]. The HPS model also embeds general health promotion philosophies throughout the approach, including principles of empowerment, holism, equity, sustainability, networking, and participation [7].

In the context of HPS, student participation can be considered a partnership or collaboration between students and various adult partners, such as teachers, school administrators, school nurses, or guidance counsellors [8]. For children and youth to be actively engaged in decisions that impact their health and development, as well as to provide them the necessary support to expand their definition of health, it is pivotal for them to be intentionally and meaningfully engaged in school health decision-making. Further, in regard to school health promotion practice, student engagement can support improvements in the physical and social environment within schools, such as building inclusive structures or developing positive relationships, as well as improving skills and competencies related to health and well-being [9].

There has been increased recognition of the importance of student engagement in HPS aligning with the philosophical shift towards the ‘new’ sociology of childhood [10]. This shift acknowledges that children and youth are active agents in constructing their lives, and are persons with capability, power, and knowledge rather than subjects of social concern. This philosophy is reflected in the adoption of the 1989 United Nations Convention on the Rights of the Child (UNCRC), a human rights treaty that provides a full list of rights for all children up to the age of 18 [11]. Adoption of the UNCRC has led to research into the benefits of student engagement in HPS, including a recent systematic review conducted by Griebler et al. [8]. This review highlighted positive outcomes of student participation in school health promotion, such as improvements in their knowledge, competence, motivation, and commitments related to health and well-being. Benefits of student engagement also expand beyond the individual and have been observed for adults and at the system-level, including improved understanding of young people, enhanced quality of their relationships with children and youth [8, 12], greater responsiveness within organizations towards the needs of children and youth within policies and programs [8] and the promotion of health equity across systems [13]. Critically engaging youth voice and dismantling power dynamics is of specific importance for children and youth who have been historically under- or mis-represented in health serving organizations such as students who identify as Black, Indigenous and People of Colour (BIPOC) and/or LGBTQIA2S+ [13–15]. Despite the increased recognition of the value of student engagement, there is still minimal focus on understanding the process factors that support student engagement in school health promotion activities [8, 16–18]. This is of relevance because student engagement is now considered a key component of a successful HPS approach [7, 19, 20].

Foundational work on the process of student engagement has been published by Jensen and Simovska through the European Network of HPS [9, 21, 22]. This work outlined the importance of creating mechanisms that facilitate and support student engagement in school health promotion to build *action competence–* the ability for children and youth to initiate and bring about positive change [22, 23]. Research has also been conducted in other jurisdictions, such as Garnett et al. [24] who analysed and interpreted publicly available data on the “Getting to the Y” project that engaged youth in their school health in the United States. Limited primary research has been identified in Canada, besides a Youth Engagement in HPS Toolkit [25] developed for practical purposes, as well as a scoping review by Beck and Reilly [26] that touched on the enablers of youth engagement. The latter identified the need for a long-term vision, youth ownership, opportunity for students to express their voice, and supportive relationships.

Although many factors contribute to the process of student engagement, to our knowledge there has not been a thorough review conducted to map and characterize the available evidence to date. A scoping review is ideal for this purpose as the main aim of a scoping review is to explore the breadth of the literature on a specific topic of inquiry, map the evidence and inform future research on a topic [27]. A preliminary search of CINAHL, ERIC, Scopus, and the JBI Evidence Synthesis database was conducted but no current or in-progress scoping reviews on the topic were identified. Though the recent review by Beck and Reilly [26] in 2017 did outline factors that promote student engagement, they did not focus on the process of student engagement, and there was limited information related to the barriers, or the characteristics of the engagement activities being conducted. This review also had a time limit of 2000–2013, only included secondary students and excluded lower levels of engagement activities (e.g., tokenism). Our scoping review used broader inclusion criteria to capture various components of the student engagement process. The purpose of this scoping review was therefore to comprehensively map and characterize the evidence on the process of student engagement in school health promotion, with a focus on whole-school health promotion models such as HPS, thereby extending the work of Beck and Reilly [26]. An open-access, peer-reviewed scoping review protocol for this work was previously published [28].

## Methods and analysis

The scoping review was conducted in accordance with the Joanna Briggs Institute (JBI) methodology for scoping reviews [29], as well as the Arskey and O’Malley five-stage methodological framework for conducting scoping reviews [30]. We used the Preferred Reporting Items for Systematic Reviews and Meta-Analysis extension for scoping reviews (PRISMA-ScR) to guide our reporting [31]. The PRISMA-ScR checklist can be reviewed in Additional File1. Definitions of the terms used in the review can be referenced in the previously published protocol [28].

## Stage 1. Identifying the research question

We conducted a scoping review to map the available evidence related to the process of engaging students in school health promotion, with a specific focus on whole school health promotion approaches, to address the following review question:

1. What is known from the existing literature about the process of engaging students in school health promotion with specific focus on whole school health promotion approaches? Additional sub-questions included:

I. What are the program/activity strategies for engagement of students in school health promotion with specific focus on whole school health promotion approaches?
II. What are the facilitators and/or barriers to engagement of students in school health promotion with specific focus on whole school health promotion approaches?
III. What is the form of student engagement in school health promotion with specific focus on whole school health promotion approaches?

## Stage 2: identifying relevant studies

We used the population/participants, concept, and context (PCC) mnemonic suggested by JBI [29] to develop our inclusion and exclusion criteria for the scoping review. The PCC is as follows:

### Participants

All sources that examine or study children and youth in any country, aged 5–19, who attended a private or public primary, middle and/or high school. Publications describing participation of other populations (university or college students, kindergarten children, teachers, principals, parents, community, etc.) were excluded.

### Concept

All sources that described the process of student engagement in school health promotion, such as the type of engagement activities, the form of engagement, and facilitators and barriers to influencing engagement, were included. Sources that only described the outcome and/or effectiveness of student engagement in the findings were excluded.

### Context

All sources that described the process of student engagement in school health promotion were included.

### Types of sources

This scoping review considered sources employing quantitative, qualitative, and mixed- and multi-methods, different forms of reviews, and grey literature sources, including dissertations and reports. As HPS is a globally recognized model, the scoping review captured evidence internationally and did not limit the search to English languages. Studies published or available in all languages were included if an English language abstract was available. Full-text data extraction only occurred if an English translated source was available. Studies published from 1986 to May 2023 were included as 1986 marks the publication date of the Ottawa Charter for Health Promotion where schools were first identified on an international stage as an optimal setting to influence student and staff health and well-being [32].

### Search strategy

In alignment with JBI recommendations, a three-step approach was taken to the search strategy. An initial search strategy was developed by the lead researcher (JCK) and peer-reviewed by a health sciences librarian using the Peer Review of Electronic Search Strategies (PRESS) guidelines [33]. The first step was an initial limited search of CINAHL on the topic. The text words in the titles and abstracts of relevant articles, as well as the key words and index terms used to describe the articles were used to develop a more comprehensive search strategy that was used for all databases included. The search strategy, including all identified key words and index terms were adapted for each of the included databases (see Additional File 2). The reference list of all full-text sources included in the review were screened for additional studies. We searched the following databases: CINAHL (EBSCO), ERIC (ProQuest), MEDLINE (Ovid), and Scopus (Elsevier). We also searched unpublished studies/grey literature including the first 50 pages of Google scholar and relevant organizations that aligned with the topic of study, such as the WHO and Physical and Health Education Canada. The search was first conducted in April 2022 and repeated in April 2023.

## Stage 3: study selection

**Fig. 1:**
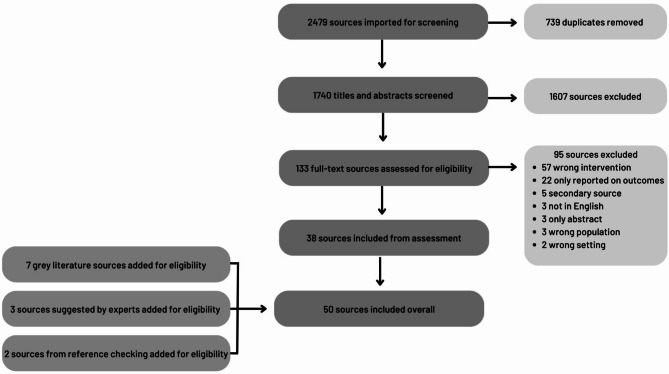
PRISMA-ScR flow diagram

Following the search, all identified citations were collated and uploaded into Covidence [34] and duplicates removed. Titles and abstracts were then screened by two independent reviewers (JCK and CM) for assessment against the inclusion/exclusion criteria. Articles were excluded if the full text was not available in English. The full texts of selected citations were assessed in detail against the inclusion criteria by two independent reviewers (JCK and CM). Primary reasons for exclusion of full-text studies that did not meet the inclusion criteria were recorded and presented in the PRISMA-ScR flow diagram (Fig. 1).

## Stage 4: charting the data

A data extraction tool was developed and used to chart the data by the lead researcher (JCK) in Microsoft Excel and was reviewed by CM and SFLK (see Additional File 3). The data extraction tool included key information for each article, as well as relevant findings related to the review questions, i.e., title; author(s); source; year of publication; journal; country of origin; type of evidence; aims/purpose; methodology/methods; study paradigm; study approach; study setting; participants; age and grade of participants; school level; sex and/or gender, ethno-racial community; geographical/social description; description of student engagement program/activity strategies in school health promotion; key findings of the study; description of facilitators and barriers to student engagement in school health promotion; and outcome of the student engagement program. Although this review excluded articles that only reported on outcomes of student engagement in school health promotion, if the articles included in the review that reported on the process of student engagement, also reported on outcomes, this was documented in the data extraction tool as secondary information and outlined in the results section.

Data extraction was first trialled from papers identified for the scoping review by two independent reviewers (JCK, CM). Reviewers met after extracting five papers each to review charting, and any discrepancies were resolved through discussion by reviewing and clarifying any confusion of inclusion/exclusion criteria, and definitions of items on the data extraction tool. After the extraction tool was finalized, full texts of selected sources were divided in half and JCK and CM used the data extraction tool to chart the data independently. JCK reviewed CM charting and HATC reviewed JCK charting.

## Stage 5: collating, summarising, and reporting the results

The charted data are presented in table and narrative form to align with the review objectives and associated questions. Descriptive statistics were conducted in Microsoft Excel for quantitative data (e.g., year of studies, geographic distribution of studies, age, and type of participants). Qualitative data (e.g., open-ended text charted as programs/activities, facilitators, barriers and outcomes) were imported into Nvivo Software for Qualitative Analysis [35]. Inductive content analysis [36] was used to identify codes and develop main categories within each charted group including program/activity strategies, and facilitators/barriers. Deductive content analysis [36] was then employed to further categorize facilitators and barriers according to the Socio-ecological Model (SEM) as individual, social or system factors [37]. Categories were defined as classifying large amounts of text into groupings that represent similar meaning through explicit or inferred communication of language [36]. JCK analyzed data charted to program/activity strategies and facilitators/barriers. Form of engagement was categorized into a 5-level scale outlined in Jensen and Simovska [22] that specifically focused on student engagement in a school health promotion context (Table 1). Relevant articles were categorized by JCK and CM in Microsoft Excel and further reviewed by HATC. As secondary information, data charted as outcomes of engagement were also analyzed by HATC.

**Table 1:** Form of student engagement (adapted from Jensen and Simovska [22])

Level	Description
5	Students suggest, common dialogue, common decisions
4	Students suggest, student dialogue, students’ decisions
3	Adults* suggest, common dialogue, common decisions
2	Adults suggest, no dialogue, students accept or reject
1	Given decisions (by adults, legislation, etc.), no dialogue, students clearly informed

*For the purposes of this research, the term ‘teachers’ was changed to ‘adults’ to encompass all adults who support student engagement in school health promotion

### Deviation from original protocol

Based on the participant inclusion age of 5–19 years that was chosen for the review, we replaced the term “youth engagement” with “student engagement” when describing the concept under study. This did not impact the search results as key words used in the search strategy already included terms such as “student,” “kid” and “children” to capture school-aged children and youth. ProQuest Dissertations and Theses Global databases were not searched independently as they were included when inputting search criteria into ERIC. The SEM was not used to deductively analyze program/activity strategies after content analysis as the narrative description of programs/activity strategies did not align with this framework format. The program/activity strategies identified in the literature mainly focused on a singular level of the SEM (i.e. interpersonal level), thereby focusing more on overall program/activity strategies for engagement rather than different levels of influence was deemed more appropriate. Lastly, Hart’s Ladder of Participation [38] was originally planned to be used to categorize the form of student engagement, but based on the content area and objectives an adapted version of the scale specific for student engagement in school health promotion was used [22] (described in Stage 5).

## Results

### Search results

After 739 duplicates were excluded, the literature search resulted in 1740 citations. Screening of titles and abstracts excluded 1607 articles, leaving 133 eligible for full-text review. Following full-text review, 38 articles were included in the study. Primary reasons for exclusion of 95 sources at full-text screening included 57 that were the wrong intervention (not focused on school health promotion), 22 that only reported on outcomes, 5 that were a secondary source, 3 that were not available in English, 3 that only comprised an abstract, 3 that included the wrong population, and 2 that were in the wrong setting.

No new sources were identified from reviewing the first 50 pages of Google Scholar. Thirty-five sources were identified from the reference searching, but after review, only 2 new sources were identified. Twelve grey literature sources were identified from related websites, and after review 7 were included. A list of websites searched can be found in Additional File 4. Three further sources were added based on expert opinion. See Fig. 1 for the PRISMA-ScR flow diagram of search results, source selection and inclusion process [31].

In summary, there were 50 sources included in the review. Additional File 5 summarizes key source information included.

### Demographics of included sources

Of the 50 sources included in the review, all were published between 2000 and 2023. Over half the studies were from European institutions/organizations (*n* = 26) [9, 17, 22, 23, 39–60] followed by North America (*n* = 18) [14, 15, 24–26, 61–72]. A small number of studies were published in other parts of the world including Asia, Africa, Australia and South America (*n* = 6) [73–78]. Most studies were primary studies (*n* = 34) [9, 14, 15, 17, 24, 39, 40, 42, 43, 45–49, 51–53, 55–59, 62–64, 67, 71–77, 79], while other sources included were commentaries (*n* = 6) [22, 41, 44, 50, 54, 61], evidence syntheses (*n* = 3) [26, 60, 78] and grey literature (*n* = 7) [23, 25, 65, 66, 68–70].

Of the primary studies, most involved only student participants (*n* = 18) [14, 24, 39, 43, 45–47, 49, 55, 57, 60, 63, 67, 71–74, 77] from a range of school levels including elementary (*n* = 11) [39, 43, 46, 49, 51, 52, 57, 64, 72, 75, 79], middle (*n* = 3) [40, 45, 55], high school (*n* = 3) [62, 66, 73] and combined (*n* = 13) [15, 40, 48, 50, 55, 58, 62, 63, 66, 67, 73, 76, 77]. Twenty sources reported on sex/gender of the participants [15, 22, 40–43, 47–49, 51, 59, 62, 64, 66, 71, 73–75, 77, 79], 17 reported on socio-racial status of the participants (e.g. race/ethnicity, socio-economic level of participants/setting) [14, 15, 40, 45, 46, 49, 57, 59, 62–64, 66, 71, 73, 75, 77, 79], and 12 reported on if the school was in a rural or urban setting [15–47, 49, 57, 64, 66, 67, 72, 74, 75, 77].

Most sources in the study used qualitative designs (*n* = 28) [9, 14, 23, 24, 39–42, 45–48, 52, 55, 56, 58, 62, 64, 66, 67, 72–77, 79], while other sources used mixed- or multi- methods (*n* = 7) [15, 49, 53, 57, 58, 63, 65], quantitative (*n* = 3) [43, 51, 71], or evidence synthesis designs (*n* = 3) [26, 60, 78]. Qualitative methods used included interviews (*n* = 22) [9, 14, 15, 17, 40, 41, 48, 53, 55, 57–59, 63, 64, 67, 72, 74–77, 79], focus groups (*n* = 21) [14, 15, 24, 39, 40, 42, 47–49, 52, 53, 58, 59, 62, 63, 65, 72, 73, 75, 76, 79], photovoice (*n* = 3) [14, 59, 77], observational techniques (*n* = 9) [17, 45, 55, 58, 64, 66, 72, 75, 79], document review (*n* = 10) [9, 15, 17, 46, 55, 58–60, 62, 66], and reflective activities (*n* = 4) [52, 59, 66, 77]. Other methods used in the sources also included survey tools (*n* = 7) [15, 43, 51, 53, 58, 63, 71] and evidence synthesis techniques (*n* = 3) [26, 60, 78]. The methodological paradigms and approaches ranged in terminology, but largely were associated with a qualitative paradigmatic view with participatory methodologies most frequently reported (*n* = 19) [14, 23, 24, 39, 41, 46, 48, 51, 52, 55, 59, 62, 66, 67, 71, 72, 75, 77, 79], as well as case study approaches (*n* = 10) [9, 23, 17, 46, 53, 55, 64, 71, 72, 76]. Table 2 outlines further demographic information on the sources included in the review.

**Table 2:** Demographics of included sources

Characteristic (n*)	Category	*n*	%
Year of publication – by 5 years (*n* = 50)	2000–2004	4	8%
	2005–2009	6	12%
	2010–2014	11	22%
	2015–2019	19	38%
	2020–2023	10	20%
Continent (*n* = 50)	Europe	26	52%
	North America	18	36%
	Africa	4	8%
	Asia	1	2%
	Australia	1	2%
Type of Evidence (*n* = 50)	Primary Study	34	68%
	Grey literature	7	14%
	Commentary	6	12%
	Evidence Synthesis	3	6%
Methodological Design (*n* = 41)	Qualitative	28	70%
	Multi- or Mixed-Methods	7	18%
	Quantitative	3	8%
	Evidence Synthesis	3	8%
Participants (*n* = 39)	Students	18	46%
	Students and Adults	16	41%
	Adults	3	7%
	Not Reported	2	5%
School Level (*n* = 40)	Elementary	11	28%
	Middle	3	8%
	High	3	8%
	Combined	13	33%
	Not Reported	10	25%
Rural/Urban (*n* = 39)	Reported	12	31%
	Not Reported	27	69%
Sex and Gender (*n* = 39)	Reported	20	51%
	Not Reported	19	48%
Methods**^**	Interviews	22	44%
	Focus Groups	21	42%
	Document Review	10	20%
	Observations	9	18%
	Surveys	7	14%
	Reflective Activity	4	8%
	Evidence Synthesis	3	6%
	Photovoice	3	6%
	Other	5	10%

*N is different for each characteristic as not applicable sources were not included

**^**Total number of methods is higher than sources included as multiple methods were used in some studies

### Program/Activity strategies for student engagement in school health promotion

From applicable sources of primary research that outlined programs/activity strategies for student engagement in school health promotion there was a range of content topics with most programs/activities having a broad focus on health (*n* = 27) [9, 14, 15, 23, 24, 26, 39, 43, 45, 46, 50, 51, 56, 57, 59, 61–63, 65, 66, 69–71, 73, 74, 77, 79], while other programs/activities focused on food/nutrition (*n* = 3) [53, 55, 72], physical activity, (*n* = 2) [42, 49], both food/nutrition and physical activity (*n* = 3) [17, 47, 64], mental health (*n* = 3) [40, 48, 58] and disease prevention (*n* = 1) [76].

Across applicable sources, categories were developed based on the common program/activity strategies to engage students in HPS. Program/activity strategies were regularly outlined in a cycle of engagement that were acknowledged as non-linear and iterative, thus are outlined in no particular order including: (1) Co-development between adults and students, (2) Opportunity to reflect and envision health concepts, (3) Developing and determining priorities through inquiry, (4) Action-oriented learning, and (5) Alignment with school focus.

An overarching category was the use of participatory approaches to enhance meaningful and collaborative engagement of students in school health promotion. This overarching concept was apparent in each of the outlined categories such that student voice was intentionally involved in different stages of the process of student engagement in HPS including development, planning and implementation [9, 17, 23, 24, 39, 43, 47, 48, 52, 63, 65, 66, 71]. Of note, eight sources identified the use of the IVAC model (Investigation, Vision, Action, Change) when outlining the program/activity strategies instilled as a guide for student engagement in school health promotion [9, 17, 22, 23, 41, 43, 55, 57]. The IVAC model encompasses various participatory principles identified in other program/activity strategies, but in a more directed manner where children and youth are supported to investigate different health issues that affect them, create visions about desirable changes, and act towards desirable action [43].

1. Co-development between adults and students.

Programs and activities that engaged students in school health promotion widely involved the co-development of projects through shared-decision making between adults and students [9, 17, 23, 24, 46, 47, 51, 58, 66]. This was regularly put into practice through the development of formal or informal committees made up of students and different adult supporters such as teachers, school nurses, administrators, and designated healthcare or health promotion professionals [46, 51, 66]. The collaborative role of the adults in said groups was largely to guide students across a participatory cycle of engagement [9, 17, 24, 48, 51, 58, 61, 62, 76], such as through facilitating group discussions [24] or aiding in data collection and analysis [61]. Adequate training was identified as a necessary component of co-development, such that it is important for both students and staff to be well-equipped with the knowledge and skills needed to meaningfully take on roles related to school health promotion activities and student engagement [24, 47, 57, 58, 62, 71].

2) Opportunity to reflect and envision concepts of health.

A strategy identified across various school health promotion programs and activities involved the opportunity for children and youth to have space and time to reflect and envision their own concepts of health [9, 14, 17, 39, 43, 45, 46, 51, 57, 59, 66, 73]. Many of the programs and activities recognized health as a broad concept that is multidimensional, multidisciplinary and a collection of personal, social and environmental connections [9, 15, 17, 23, 43, 51, 55, 57–59, 62, 73]. Thus, this ideology translated into programs/activities that provided the opportunity for students to subjectively develop their own perceptions of health through personal and collective experiences with peers [39, 45]. Methods to evoke reflection and visions of health included reflective writing [73], facilitated dialogue [24, 39], and taking and interpreting photos of the school health environment [14, 59].

3) Developing and determining priorities through inquiry.

An additional strategy noted in various school health promotion programs and activities involved students assessing their individual and school health needs and further developing priorities based on their inquiry [17, 24, 40, 46, 53, 57, 59, 62, 66, 71]. Priority decision making involved acts of strategic planning, expressions of needs and deciding on issues affecting their lives through different data collection and analysis techniques [14, 23, 24, 40, 49, 52, 58, 59, 61, 63, 66, 71]. Involving students in inquiry allows them to further examine the health needs of their peers and/or wider school community [40, 58, 64, 66, 71], as well as increases their ownership through understanding of their own health data [24]. Methods executed in the sources included employing needs assessments and surveys [71], conducting photovoice projects [14, 59, 61] and peer to peer interviews [67].

4) Action-oriented learning.

Action-oriented learning was a key characteristic of engaging and sustaining students in school health promotion programs and activities [9, 17, 23, 24, 39, 41, 43, 46, 53, 57, 66, 71, 72]. In practice, this involved students recognizing the ability to enact change through connecting their reflective, visionary and priority exercises to realistic action in their school health environment [39]. Sources identified that this connection from school health promotion theory to action can be gained through participation in practice-based mechanisms such as presenting their vision, advocating for change and promoting external dialogue on issues of interest [23, 24, 57, 59, 66]. Further, being involved in the operations of actioning ideas such as mobilizing resources or participating in on the ground implementation [53, 65, 66, 72] was essential for students to link vision to change.

5) Alignment of strategies with school focus.

A distinct tactic employed by programs and activities aiming to actively engage students in an initiative or project related to school health promotion was to strategically embed and/or align their objectives with pre-established practices in the school curriculum [17, 45, 47, 49, 51, 55, 62]. For example, this often showed up as embedding health promotion initiatives such as physical health as an ongoing part of different school classes including math or language arts [49, 51] or attaching a certain health promotion program or project to a pre-developed health class such as Health Education [55, 57].

### Facilitators and barriers to student engagement in school health promotion

**Fig. 2:**
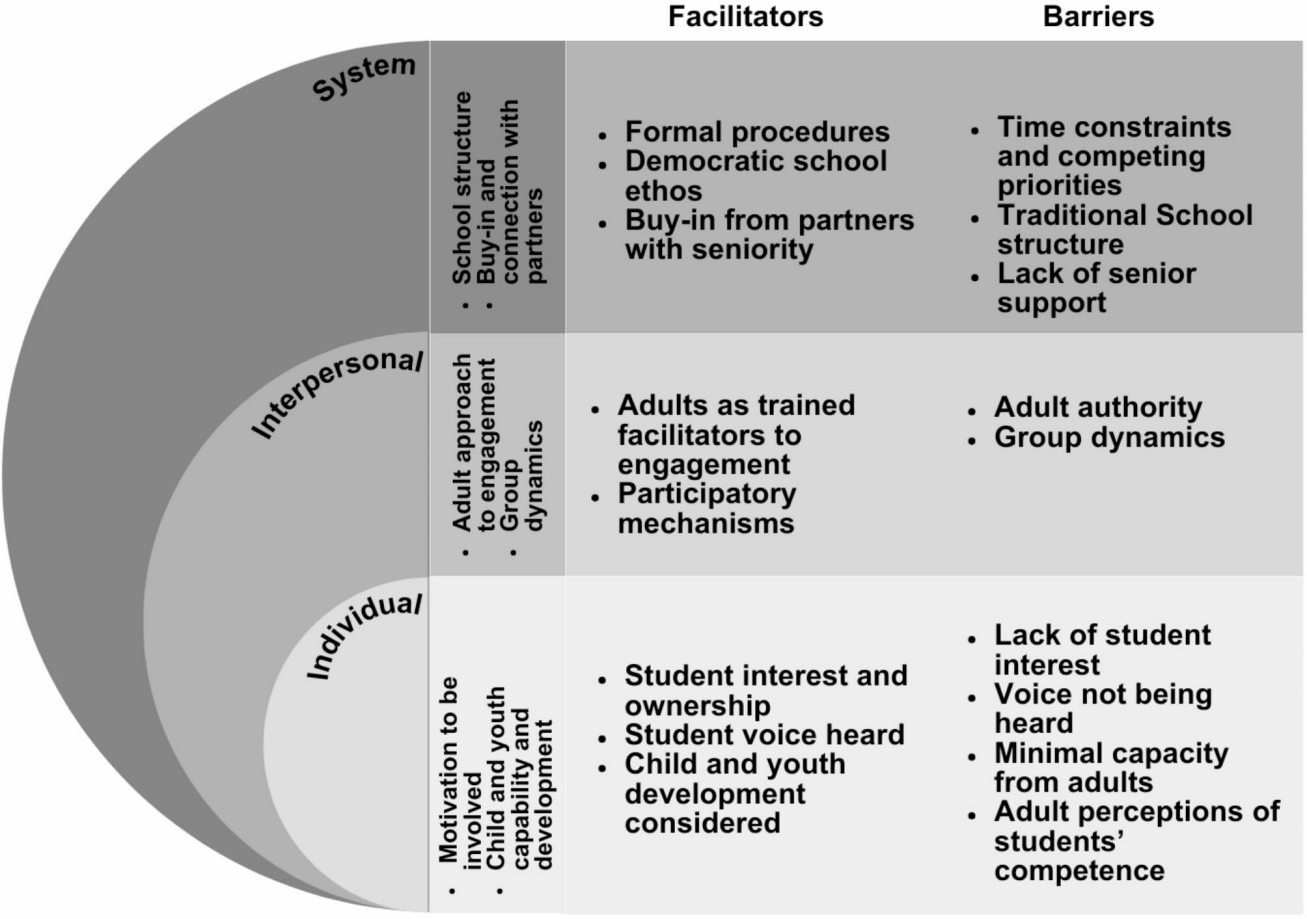
Facilitators and barriers to student engagement in school health promotion categorized by the socio-ecological model (SEM)

Intersecting facilitators and barriers to student engagement in school health promotion were identified at each level of the SEM including individual, interpersonal, and system-level factors. The facilitators and barriers were found to be overlapping; therefore, categories were developed to capture both promoting and hindering factors to the engagement process for contextual purposes including: (1) Student and adult motivation to be involved, (2) Child and youth capability and development, (3) Adult’s approach to engagement, (4) Group dynamics, (5) School structure, and (6) Buy-in and connection from partners with authority. To aid with clarity and deviation of influential factors, facilitators and barriers are further broken down by the SEM in Fig. 2.

*Individual-level*.

1. **Student and adult motivation to be involved**: Sources suggested that overall student motivation such as their wants, needs and desires can impact their degree of engagement in school health promotion. Their interest and commitment were found to be either a promoter or deterrent to their involvement and was largely linked to their feeling of ownership [17, 22, 24, 25, 41, 44, 49, 60, 62, 66, 67, 72, 75, 79] or lack thereof [22, 23, 26, 39, 41, 67, 76]. Student ownership was manifested as having their opinions taking seriously [26, 44, 47, 51, 60, 67, 70, 75, 76] versus their voice not being heard [22, 23, 26, 39, 41, 67, 76]. Student involvement in understanding their own data can heighten the ownership process [48], as well as peer-to-peer support [15, 40, 41, 44, 46, 47, 64, 70, 72, 73, 75, 79] such as dialogue and collaboration between pupils and student mentoring. In relation to adults, the capacity to be actively involved in engagement practices is a limitation, such as the energy it takes to meaningfully facilitate and support students, as well as the commitment to long-term reform [23, 53, 61, 62].
2. **Child and youth capability and development**: It was recognized from the sources that a facilitator to engagement was when adults recognize student capability and consider their level of cognitive development to be of value, rather than a limitation to engagement [25, 26, 54, 61, 62]. In practice, this translates to a scaffolding approach where adults meet students where they are at through youth friendly materials [25, 79] or use of methods consistent with the development of logical reasoning [61]. By contrast, a barrier noted was the pre-conceived attitudes and perceptions that adults can hold regarding the limited capability and competence students need to be actively engaged in school health promotion [23, 25, 41, 45, 50, 54, 59, 61, 72, 76]. These perceptions can lead to adults adapting what is ‘appropriate’ to teach in class [45] or limiting engagement opportunities for students [41, 72]. As stated by Vanner et al. [72, p. 342] engagement is often viewed as an “instrumental perspective of participation rather than a child rights conception,” leading to tokenistic rather than genuine involvement [41, 72].

#### Interpersonal-level

3) **Adult’s approach to engagement**: The practical approaches taken by adults to engage students in school health promotion have considerable impact on promoting or hindering engagement. Adults who approach engagement through the role of a facilitator such as supporting, guiding and encouraging student voice can enhance the engagement process, rather than top-down teaching mechanisms [15, 23, 40, 44, 67, 71, 73, 79]. This form of support was referenced as adults being open-minded to collaborate with students [9, 26, 40, 54, 60–62, 79], build trusting relationships [14, 25, 40, 41, 61, 74, 77, 79], encourage students to participate [15, 40, 44, 67, 73], and applying participatory mechanisms [9, 17, 24, 39, 46, 51, 66, 70]. The practice of participatory mechanisms largely related to the use of experiential learning to actively engage students with their peers and the social/physical health environment [17, 24, 40, 43, 49, 64, 73, 75, 76] such as ‘learning by doing’ [75] and practice-based activities [49]. However, it was also identified that for adults to engage students adequately and appropriately, they must have the capability to do so through capacity building and training opportunities [17, 22, 44, 59, 60, 72–75].

Approaches to engagement that mimicked authoritative and hierarchal practices negatively impacted the process of student engagement in school health promotion. This includes the act of adults driving decisions [53, 59, 62, 69, 77] such as monopolizing discussions [62] and a lack of shared decision-making with students [9, 17, 48, 59, 62]. The sources also identified that adults can feel a ‘loss of control’ during more collaborative practices; thereby further inhibiting meaningful engagement with students [25, 44, 75, 76].

4) **Group dynamics**: Group dynamics mainly related to the structure of a formal or informal group or committee influencing the engagement of students in school health promotion. Composition of the group including diversity, and number of students as well democratic election processes, if applicable, impacted the level of engagement [55, 60, 61, 67, 75]. For example, larger group sizes were viewed as a barrier to engagement practices [55, 61, 75]. Further, lack of clarity about student/adult roles were shown to impact engagement [15, 48, 58], such as what responsibilities were expected of the students versus adults in facilitating activities and leading tasks [48, 58, 60]. This confusion also impacted relationship dynamics between students and adults such as power relations and ability to collaborate [17, 48, 59, 62]. Of example, adults have more focus on outcome measures compared to students, a phenomenon which can inadvertently impact the participatory process [17, 48].

#### System-level

5) **School structure**: The structure of the school such as its overall culture, ethos and policies impacts the engagement of students in school health promotion. It was acknowledged by sources that schools which promote democratic, reflective and supportive practices enable authentic participation of students [17, 22, 54, 60, 61, 75, 78]. Examples related to this ideology include collaborative dialogue between students and teachers, rather than imposing participation of a task on a student [22], as well as providing a system of support for adults to build capacity in HPS and participatory approaches [78]. It was also recognized in the sources that an enabling factor is if organizational principles and beliefs are formalized into regulated or guiding policies on student engagement in HPS. Examples include developing structured timelines, formal development of action teams, building boundaries of roles/responsibilities for student and adult partners (e.g. terms of reference), and clearly defining the importance of student voice in administrative documents related to school health promotion [40, 53–55, 60, 62, 71, 72, 78, 79].

By contrast, a number of sources highlighted that a school’s more traditional and historical focus on a structured and controlled environment was not conducive to meaningful engagement in school health promotion [17, 22, 23, 39, 45, 61, 62, 65, 75]. Of note, traditional teaching methods that centre students as recipients of information, rather than reflectors or producers of knowledge, was identified as a barrier [14, 17, 22, 41, 45, 54, 59, 62, 65] such as maintaining students in their role as pupils and “subordinates” to teachers [39, p. 25]. In relation to school health promotion, this involves students being taught and made to accept pre-existing health ideologies of what is determined ‘healthy’ or not, versus open dialogue and reflection on what they believe to be related to their health and well-being [54]. The value and pressure placed on improvement outcomes such as academic achievement practices [9, 15, 45, 62] were also acknowledged to detract from student engagement in school health promotion practices. Lastly, time constraints due to competing priorities of the traditional school structure such as extracurriculars, an overloaded curriculum, and administrative duties [15, 17, 23, 26, 45, 50, 53, 58, 61–63, 65, 71, 72, 75, 76] also served as a barrier to engagement.

6) **Buy-in and connection with partners with authority**: Buy-in and connection with partners who have authority [17, 23, 24, 40, 58, 60, 69, 71] or lack of [17, 40, 59, 62] was noted as both a facilitator and barrier to student engagement in school health promotion. Enabling factors focused on the support of senior management [58] or principals [40] who had the ‘power’ to change wider policies and action change, while mitigating factors related to absence of senior management and partners within and beyond the school. Lack of system-level support involved absence of formal agreements, as well as minimal connection to decision-makers, or external community members who could influence the engagement process [17, 40, 59, 62].

### Form of student engagement

**Fig. 3:**
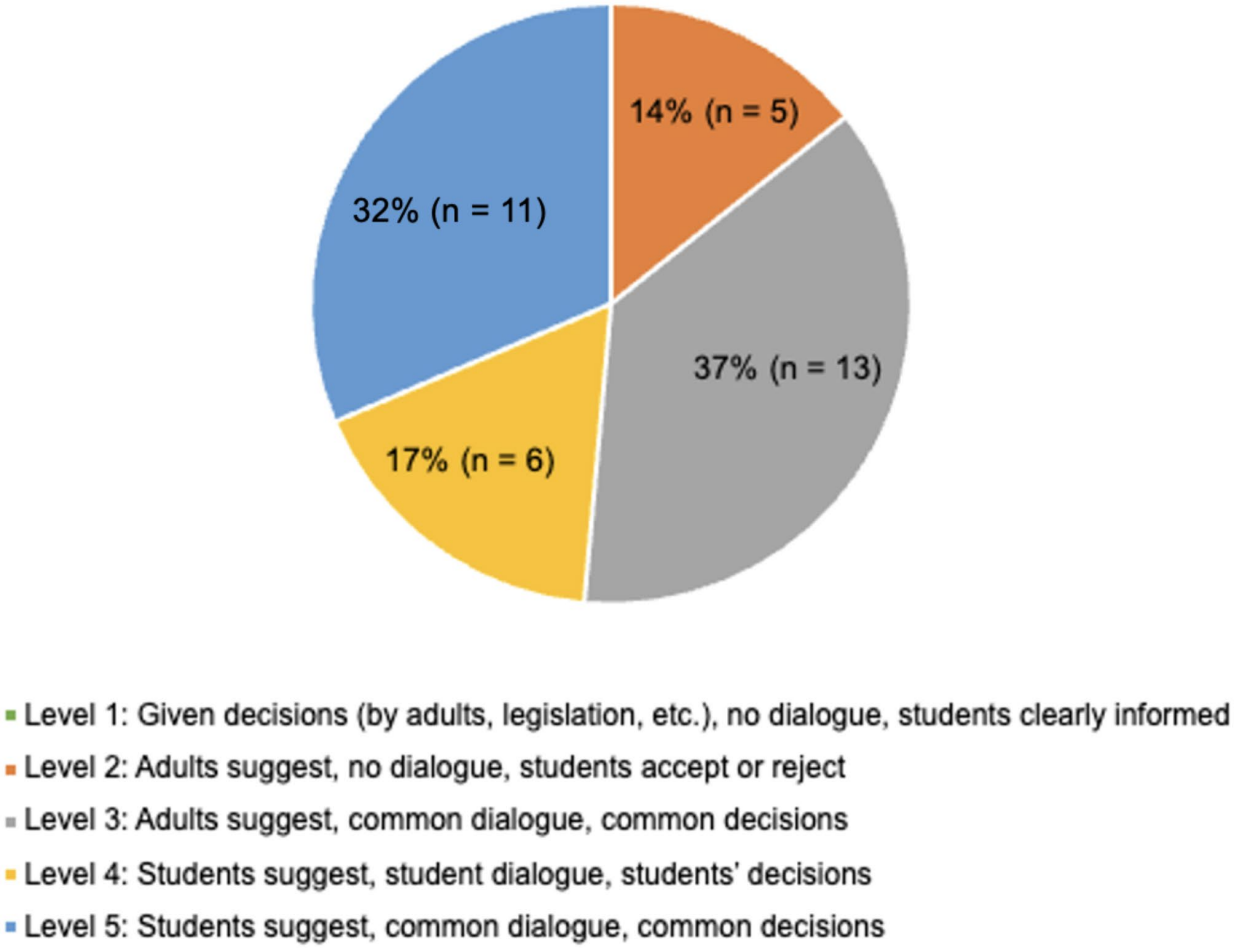
Forms of student engagement adapted from Jensen and Simovska [22]

Applicable sources (*n* = 35) ranged in their forms of engagement with most sources (*n* = 30, 86%) being categorized as Level 3 or higher (Fig. 3). Few sources were categorized at Level 2 (*n* = 5, 14%) [42, 48, 72, 73, 76] and none at Level 1. Author reasoning for categorization into specific levels of engagement can be viewed in Additional File 6.

### Outcomes of student engagement in school health promotion

As a secondary information, outcomes were included and categorized per SEM level if reported in the sources. Of the outcomes identified for engagement of students in school health promotion programs/activities, a large number of factors were reported at the individual level including enhancing student health and well-being [15, 22, 25, 43, 47, 51, 58, 73, 76], advancing personal development skills [8, 14–17, 25, 44, 47, 48, 50, 51, 54, 61, 62, 64, 66, 76], increasing student participation [15, 44, 47, 49–51, 56–59, 61, 72, 73, 77], and improving student perceptions of schools [39, 40, 43, 45, 46]. At the interpersonal level, positive peer to peer relationships were identified [9, 25, 59, 71], as well as student-adult relationships including student to teacher [25, 54, 60, 71] and student to health professional [66, 74]. Lastly, at the system-level various positive outcomes were highlighted including supporting a healthier school environment [17, 22, 39, 59, 60, 72], aiding in better health services for student’s needs [71, 74] and student-centered policies at the school level [17, 22, 25, 40, 60].

## Discussion

This scoping review mapped and characterized the process of student engagement in school health promotion, with a focus on whole school approaches. To understand the process of student engagement in school health promotion, mapping of key strategies that programs/activities apply, as well as the facilitators and barriers to the process of student engagement in school health promotion was conducted. The program/activity strategies used to meaningfully engage students in school health promotion largely related to participatory mechanisms including co-development between students and adults, opportunity to reflect and envision concepts of health, determining priority focus through student inquiry, and implementing action-oriented learning. Overlapping facilitators and barriers included influential factors related to student and adult motivation, perceptions of student capability, adult approaches to engagement, group dynamics, impact of school structure, and buy-in from system-level partners.

### Overview of source demographics

Of the 50 sources included, all were published after 2000, indicating the lack of research focus on this topic until years after the development of the 1986 Ottawa Charter for Health Promotion [32], the ratification of the 1989 UNCRC [11] and the first WHO Health Promoting Schools guidelines produced in 1995 [80]. This insight aligns with the broader literature, such as that of Caraballo et al. who noted that the use of the term ‘Youth Participatory Action Research’ (YPAR) can be traced to the year 2000 in the academic literature. Further, many sources included in the review were from European organizations/institutions, with over half of those being published in Nordic countries. As these countries are well recognized for their democratic thinking, this finding speaks to the synergistic alignment of student engagement with egalitarian practices and how vital social and environmental influences are to authentic child and youth engagement.

### Program and activity strategies

Program and activity strategies implemented for student engagement widely fell within the realm of participatory mechanisms for engagement, such that children and youth were involved in different stages of programs/activities from priority setting to implementation of ideas into action. The majority of these programs and activities adopted a broad concept of health and largely focused on school health promotion as a whole rather than specific content areas (e.g., nutrition). This finding is unsurprising due to the focus of the review, yet still reiterates the importance of adopting whole-school health approaches like HPS that view health as a dynamic concept made up of various and interconnected factors at the individual, interpersonal and system-level.

Specific strategies to ensure participatory approaches were intentionally implemented across each stage of a program or activity related to school health promotion was evident across the sources. For example, reflection and visionary practices were frequently mentioned in the sources included [39, 43, 46], as a strategy to ensure students had space to brainstorm and imagine their own conceptions of what health and well-being meant to them. Clausen et al. [39] used an approach entitled the ‘Future Workshop’ to elicit children’s concerns related to daily life and reflect on better health and well-being in the future. This approach was strategically implemented over three stages (Critical Phase, Visionary Phase, and Realistic Phase) to provide students with the opportunity to express health issues of concern, brainstorm processes for better health, and action their visions into realizable change.

Action-oriented learning that enabled student engagement to lead to real-world change was also a common practice identified in the sources [22, 39, 71]. In relation to health promotion, sources often highlighted action-oriented learning as a critical phase to enable students to conceptualize how health and well-being go beyond the individual and are interconnected to their social and physical surroundings [22, 23, 71]. An example of this foundational concept was operationalized by Soleimanpour et al. [71] who outlined two case studies of student action groups that were established to enhance school-based health service policies in Alameda County, California. The project provided training to support youth in identifying and researching the health needs of their peers and advocating for improvements in their school-based health centres including revisions of sexual and mental health policies.

Although the majority of sources were grounded in participatory processes, few studies outlined the use of specific frameworks to guide their implementation. While linear processes and direct guidance can be viewed as implanting more positivistic viewpoints on participatory and flexible practices, one framework readily referenced was the use of the IVAC (Investigation, Vision, Action, Change) approach. The IVAC approach aims to ensure meaningful involvement of students throughout school health promotion activities by providing more direct guidance of how to bridge the gap between students’ visions for health and realistic action [22]. Of note is the Young Minds web-based project implemented across eight European countries [23]. In this project, the IVAC framework was used to intentionally communicate and explore links between youth, culture, health and environment as a basis for action and change. The ‘Young Minds’ approach was used as a way of authentically involving young people in the process leading up to their participation in the WHO’s Fourth Ministerial Conference on Environment and Health: The Future for Our Children.

### Facilitators, barriers and outcomes related to student engagement

There were a number of intersecting facilitators and barriers identified in the literature that impacted the process of student engagement in school health promotion. As these influential factors were broken down by the SEM, it was notable that factors at the system-level may have top-down influence at the interpersonal and individual-level. For example, a traditional school structure that historically positions students as recipients of information [39] may be directly connected to adults’ pre-conceptions of student competence [45] and adults’ reasoning for driving decisions rather than implementing student-led initiatives [53]. Simovska et al. [41] identified that this is specifically true in the topics related to health such as smoking, alcohol and HIV that already have an overload of pre-defined information related to the area, thus limiting students’ ability to reflect and form their own opinions or gather their own information on issues related to the topic at hand. Further, facilitating factors at the system-level, including formal procedures, may have the potential to enhance engagement as indicating a statement of intent for student engagement in school health promotion can aid in accountability and monitoring of practice. Implementation of formal procedures was explicitly identified as a main finding in Tomokawa et al. [78] document review, such that having a clear indication of the importance of student participation in legal and administrative documents was a necessity for sustained uptake and engagement of students in HPS. Broader literature on HPS also suggests that the development and implementation of system-level policies and guidelines is fundamental for initiating change across school structures [81].

In relation to the interpersonal-level, the student-adult relationship was identified as a key facilitating factor such that adults who approach engagement with an open mind [26, 40, 60], encourage student participation [44, 60, 67], and are adequately trained in the field [22, 44, 59] can positively impact the engagement process. Similar to the interplay of factors at the system-level, the relationship between adult and student could have impact on a student’s motivation for involvement as their engagement relies heavily on ensuring the processes implemented allow for student ownership, promote student interest using hands-on activities [49, 75] and student-friendly inquiry (e.g. data collection and analysis) [24, 49]. Having adults who are well-versed in student engagement and HPS principles is essential to ensure there is intention and genuine actions behind the strategies implemented rather than the steps of engagement turning into a checklist or the act of engagement being tokenistic. Opportunities for training and capacity building for adults to enhance their competencies in the area is essential such as WHO’s technical reports and briefs on HPS topics [82] or UNICEF’s training on Adolescent Participation and Civic Engagement [83]. As HPS is a multi-dimensional model, it can be helpful to use frameworks like the SEM to understand the interplay of facilitators and barriers at different levels of influence and how their interconnectedness can impact one another.

Lastly, the outcomes of student engagement in HPS identified in this review reinforced those of Griebler et al. [8]. The review further highlighted the benefits of student engagement for child and youth development, and the need for adult and organizational relationships that understand and enhance the capability of students. Strengthening the evidence base for the widespread benefits of student engagement and the positive outcomes of these practices is important to ensure that HPS activities fully embed meaningful student engagement principles at all times.

### Summary and implications of the scoping review

There is a tendency within research and practice to want to know the outcomes and effectiveness of a program, initiative, or strategy prior to fully understanding the implementation factors that play a critical role in their success. Regarding HPS, this is specifically evident when it comes to understanding *how* and in what *way* students are involved in school health promotion. Although the systematic review by Griebler et al. [8] detailed the effectiveness of student participation in school health promotion, the factors that impact the process and level of engagement remain poorly understood. Our scoping review mapped and characterized the available and current literature on this topic to provide a comprehensive overview across the global literature on the process factors, including the strategies, as well as facilitators and barriers, that impact student engagement in school health promotion. Our scoping review identified that strategies for engagement largely relate to the use of participatory mechanisms at each stage of the process, yet beyond IVAC there are no specific frameworks or models that are distinct to student engagement in school health promotion practices. This is a limitation to the research area as the scoping review showcased that models specific to student engagement in school health promotion are warranted based on findings that may be unique to student engagement within a school health promotion context. For example, how meaningful engagement can be used to build student competencies related to health promotion principles or how opportunity for reflection and envisioning is pivotal for students to develop their own concepts of health. Further, the findings outlined that facilitators and barriers to student engagement show up at differing levels of influence, be that top-down factors such as school structure or bottom-up factors such as student and adult motivation for engagement. These findings reiterate the need to fully examine the scope of factors influencing the process to truly understand what is impacting meaningful student in school health promotion.

The implications of the scoping review are widespread for practice and future research. By mapping and characterizing the current knowledge on the subject provides practitioners and researchers alike a more comprehensive understanding of the process factors that influence student engagement in HPS, as well as what factors can promote or mitigate this process at the individual, interpersonal and system-level. Although the weight and importance of each strategy, as well as facilitators and barriers to engagement are assumed to vary based on school and jurisdictional context, the review provides foundational and detailed information to further recognize the essential processes needed for meaningful engagement to occur. Additionally, this work can be used to compare and contrast global findings to local operations for further reflection and adaptation. Future research may consider using these findings to help develop and refine frameworks and tools related to student engagement in HPS to build a strong theoretical understanding. Further, as differences in demographic factors including race/ethnicity, sex/gender, geographic location and income can impact the nature and level of resources available for engagement practices, this research could be built on by further breaking down the findings to examining diversity in engagement based on different socio-economic groupings. Overall, this scoping review contributes to a growing knowledge base in research and in practice on the understanding and importance of meaningful student engagement in school health promotion.

## Strengths and limitations

This scoping review followed rigorous established methodological guidelines [28–30] to help guide and report the findings, with further support from a health sciences librarian. Despite our comprehensive approach, the review has some limitations. First, the search was limited to the databases included in the review, as well as English-language for full text articles. Second, although a range of search terms and adaptations of syntax for databases were conducted, there are varying terms used to describe school health promotion and HPS across disciplines. Therefore, we may have missed some publications based on the pre-determined boundaries of this scoping review during protocol development. Third, while we focus on identifying unpublished/grey literature for the review, we may not have captured all reports or dissertations aligned with the topic of inquiry.

## Conclusion

This scoping review provides an overview of the process factors related to student engagement in school health promotion. As understanding of this topic of inquiry grows, our review offers insight into the strategies used to meaningfully engage students in school health promotion, as well as the facilitators and barriers to this process. We conclude that participatory mechanisms to engage students as active agents within school health promotion are essential. Implementing these approaches needs to take account of an array of interconnecting factors that can impact this process. Of note, adults that approach student engagement through the role of a facilitator or support can enable engagement, while school structures, such as a controlled environment and traditional teaching mechanisms, can constrain student engagement. Our findings provide insight and further guidance on factors to consider when developing strategies to engage students in a meaningful manner throughout school health promotion programs, activities, and initiatives.

## Electronic supplementary material

Below is the link to the electronic supplementary material.

Supplementary Material 1

Supplementary Material 2

Supplementary Material 3

Supplementary Material 4

Supplementary Material 5

Supplementary Material 6

## Data Availability

The datasets generated and/or analysed during the current study are not publicly available but are available from the corresponding author on reasonable request.

## Abbreviations

HPS: Health Promoting Schools
JBI: Joanna Briggs Institute
PRESS: Peer Review of Electronic Search Strategies
PRISMA-ScR: Preferred Reporting Items for Systematic reviews and Meta-Analyses extension for Scoping Reviews
PCC: Participant, Concept, Context
UNCRC: United Nations Convention on the Rights of the Child
SEM: Socio-ecological Model
WHO: World Health Organization
