# Supplementary figures and images for: The process of student engagement in school health promotion: a scoping review
Source: BMC Public Health. 2025 Mar 19;25:1063. doi: 10.1186/s12889-025-22121-8 (PMC11921550; doi:10.1186/s12889-025-22121-8)

**Additional File 2. Example of Search Strategy (CINAHL)**


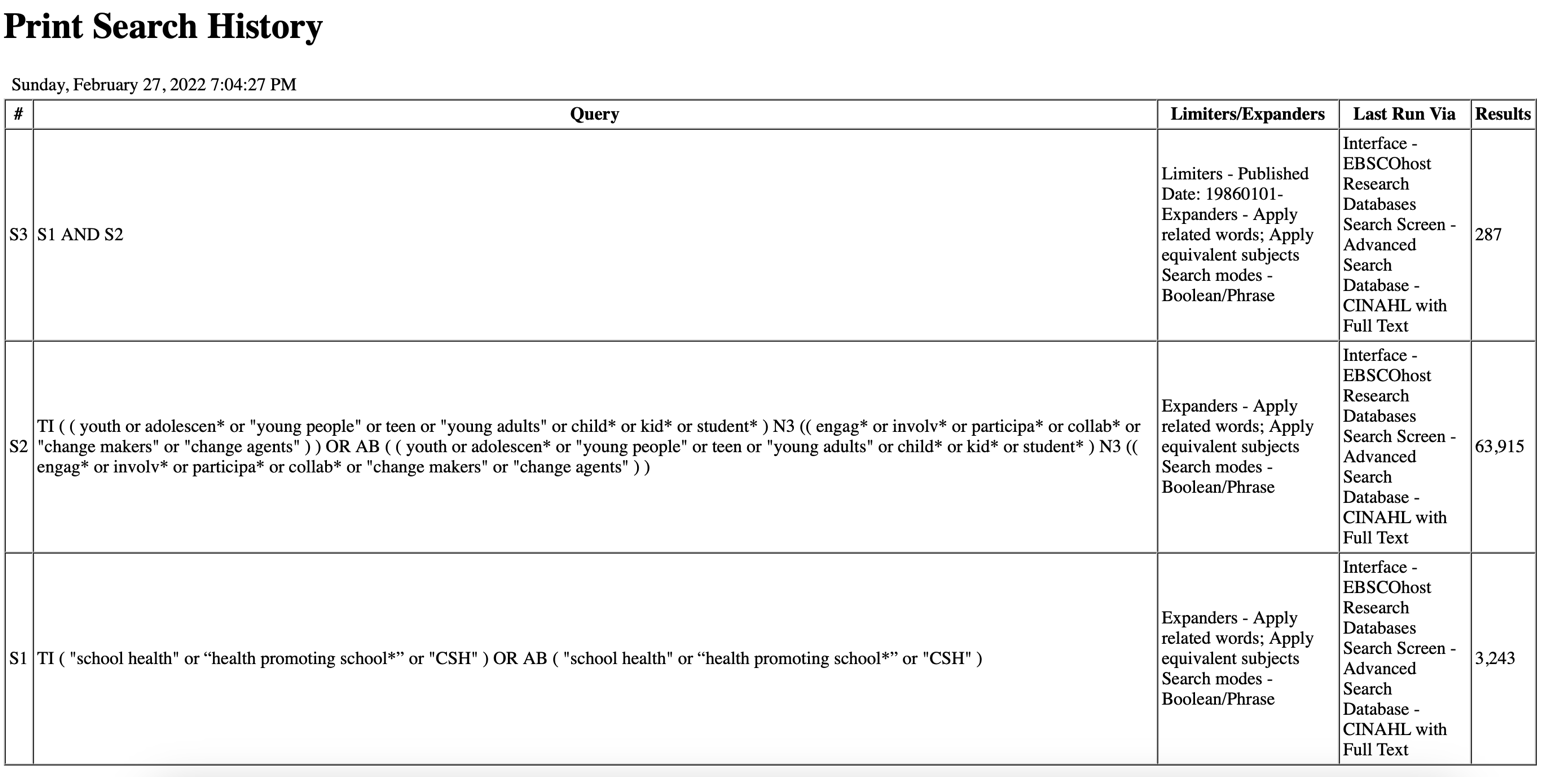

Supplement: Supplementary file 2 — Supplementary Material 2 [file 12889_2025_22121_MOESM2_ESM.docx]
