# Supplementary material for: The process of student engagement in school health promotion: a scoping review
Source: BMC Public Health. 2025 Mar 19;25:1063. doi: 10.1186/s12889-025-22121-8 (PMC11921550; doi:10.1186/s12889-025-22121-8)
Supplement: Supplementary file 3 — Supplementary Material 3 [file 12889_2025_22121_MOESM3_ESM.docx]

**Additional File 3. Data Charting Tool**

| **Evidence source details and characteristics** | |
| --- | --- |
| Citation details (e.g., author/s date, title, journal, country): |  |
| Type of evidence source: |  |
| Methodology (Paradigm, approach, design): |  |
| Method (Data tools): |  |
| Context details (e.g., setting, elementary/middle/high school, rural/urban, low/medium/high income community): |  |
| Participant details (e.g., number, age, grade, sex/gender, ethno-racial information): |  |
| Research question or study purpose: |  |
| Key findings: |  |
| **Details/results extracted from source of evidence (in relation to the concept of the scoping review)** | |
| Program/intervention/activity strategies for student engagement in school health promotion |  |
| Barriers to student engagement in school health promotion |  |
| Facilitators to student engagement in school health promotion |  |
| Outcome of student engagement in school health promotion |  |
