# Supplementary material for: The process of student engagement in school health promotion: a scoping review
Source: BMC Public Health. 2025 Mar 19;25:1063. doi: 10.1186/s12889-025-22121-8 (PMC11921550; doi:10.1186/s12889-025-22121-8)
Supplement: Supplementary file 4 — Supplementary Material 4 [file 12889_2025_22121_MOESM4_ESM.docx]

**Additions File 4. List of Websites searched for Grey Literature Sources**

| **Title** | **Website** |
| --- | --- |
| Pan-Canadian Joint Consortium for School Health | http://www.jcsh-cces.ca |
| School Based Health Alliance | https://www.sbh4all.org |
| Society for Public Health Education (SOPHE) | https://www.sophe.org |
| World Health Organization | https://www.who.int |
| Youth Engagement in Health Promotion | https://jps.library.utoronto.ca/index.php/youthengage/about |
| Act for Youth | https://www.actforyouth.net |
| Schools for Health | https://www.schoolsforhealth.org |
| Youth Engagement in Health Promotion Powerpoint | https://jps.library.utoronto.ca/index.php/youthengage |
| UpLift Partnership | https://www.upliftns.ca/ |
| Physical and Health Education Canada | <https://phecanada.ca/> |
