# Supplementary material for: The process of student engagement in school health promotion: a scoping review
Source: BMC Public Health. 2025 Mar 19;25:1063. doi: 10.1186/s12889-025-22121-8 (PMC11921550; doi:10.1186/s12889-025-22121-8)
Supplement: Supplementary file 5 — Supplementary Material 5 [file 12889_2025_22121_MOESM5_ESM.docx]

| **Additional File 5. Summary of Included Sources** | | | | | | | | |
| --- | --- | --- | --- | --- | --- | --- | --- | --- |
| **Author** | **Purpose** | **Methodology (Paradigm, Approach, Design)** | **Method** | **Setting** | **Participants** | **Age (Grade)** | **Program /Activity Strategies** | **Key Findings** |
| Abildsnes et al., 2015 | To explore physical education teachers’ and public health nurses’ perceptions of how to facilitate and promote students’ participation in class. | Pre- and post- intervention design | Qualitative: 1) Focus groups with Physical Education teachers and school nurses (pre-intervention), focus groups with Physical Education teachers (post intervention) | Two high schools in Kristiansand municipality, Vest-Agder County in southern Norway. | Adults: Pre-implementation Focus Group 1: Physical Education Teachers (n = 6)  Pre-implementation Focus Group 2: Public health nurses (n = 8) representing 4 different high schools | NR* (NR) | New model of physical education with two alternative programs: 1) Sports enjoyment, 2) Motion enjoyment. | Participants related the student's competence and attitude towards participation in physical education class to previous experiences in junior high school, to the competence of physical education teachers, and to possibility for students to influence the content of physical education programs. |
| Beattie et al., 2023 | To formally evaluate student and individual level outcomes in terms of Getting to Y: Youth Bring Meaning to the Youth Risk Behavior Survey (GTY) initiative in five domains of positive youth development and Youth Participatory Action Research (YPAR) initiatives: health literacy, community engagement, self-efficacy, knowledge and skills, and resilience and protective factors. | Convergent parallel mixed-methods design | Quantitative: 1) Survey with youth leaders  Qualitative: 1) Focus groups with students from participating school teams, 2) Written feedback from four different school teams | 20 Vermont middle/high schools. | Survey participants: 256 students from 20 Vermont middle/high schools [youth leaders (n = 196) or data analysis retreat participants (n = 64)],  focus group participants: n = 50 students | Age: NR  Survey participants: NR  (Focus Group participants: 2 (Grade 5), 3 (Grade 6), 10 (Grade 7), 8 (Grade 8), 1 (Grade 9), 2 (Grade 10), 3 (Grade 11), and 2 (Grade 12).  Written Feedback Participants: 8 (Grade 7), 11 (Grade 8), 5 (Grade 9), 4 (Grade 10), 6 (Grade 11), 3 (Grade 12). | Getting to Y: Youth Bring Meaning to the Youth Risk Behavior Survey (GTY): A positive youth development initiative, whereby students analyzed local youth health data and created change. | Statistically significant improvements were shown in all five domains from pre- to post- intervention surveys and differences in effect by gender. Focus group findings also indicated participation in GTY positively affected participant’s understanding of their health and well-being and increased agency. |
| Beck & Reilly, 2017 | To review student engagement in health promotion with a focus on secondary students’ initiatives in school settings. | Scoping review | Synthesis: Scoping review | Adolescents in secondary school without clinical or developmental disorders. | NA^ | Adolescents  (NR) | Any extracurricular activity that encouraged student engagement outside the traditional academic curriculum and took place within the school community. | The scoping review indicated four main themes including: 1) Engaging students in health promotion can advance their sense of belonging in a community, 2) Encourage meaningful involvement, 3) Give voice to student concerns, and 4) Advance supportive and dynamic relationships. |
| Boberova et al., 2017 | To examine whether action-oriented teaching, using the Investigation-Vision-Action-Change (IVAC) didactic model, built on the concept of children's health literacy, particularly around citizenship, improves children's well-being. | Cluster-randomized controlled trial | Quantitative: 1) Survey by students (pre- and post-) | Five elementary schools based in the eastern region of Slovakia. Within each school, two fourth grade classes were randomly selected, and randomly assigned the experimental treatment (intervention program) to one of them. | Students: total (n = 180); experimental group (n = 87); control group (n = 93) | 9 -11  (Grade 4) | Voices for Health: An intensive course spanning over 16 weeks. This intervention program employed the Investigation-Vision-Action-Change (IVAC) model for action-oriented teaching, where children were supported to investigate different health issues that affect them, create visions about desirable changes, and  act toward desirable change. | The intervention program was shown to improve children’s perceptions about school, violent behavior, and their well-being. |
| Boonekamp et al., 2021 | To unpack the concept of agency by drawing on insights from the Capability Approach. This provides input for the integration of agency in Health Promoting Schools and salutogenic approaches, to enhance physical activity-related agency. | NA | NA | NA | NA | NA  (NA) | NA | The authors argue that an agency perspective provides an important contribution for physical activity and salutogenic health promotion strategies at school. |
| Bruselius-Jensen et al., 2014 | To explore how pedometers can be used as tools in participatory health education to enhance primary school children’s insights into, and abilities to reflect on, physical activity in their everyday practice. | NR | Mixed methods: 1) Self-reported step counts of students, 2) Focus groups with students. | Four primary schools from different parts of Copenhagen, Denmark. | Students: (n = 74) | 12 -13 years  (Grade 6) | Imove: Part of a Health Promoting Schools  program named “Move@School” that aimed to integrate health education and physical activity into the formal school curriculum. | Two themes were identified when understanding the use of pedometers to enhance children’s relation to physical activity in daily practice including:  1) The exploratory design and the interaction with the pedometers seemed to promote pupil participation and, 2) The step data seemed to support pupils' abilities to reflect on daily practice. |
| Clausen et al., 2019 | To describe children’s perceptions and visions for a healthier social and physical environment in a primary school, as well discuss advantages and challenges of involving children in decision-making processes. | Everyday life perspective, Participatory Action Research | Qualitative: 1) Two workshops (focus groups) using 'social imagination' phases with students | The present sub study of Project SoL (An abbreviation of “Sundhed go Lokalsamfund in Danish or “Health and Local Community in English’)  was conducted in 2012–13 in a public primary school in the town of Nexø, Denmark. | Students: (n = 50) | 8 - 9  (Grade 2) | The future workshop was designed as a participatory forum for expressing concern and dissatisfaction with everyday life issues as well as ideas and visions for better living. Sessions included three phases: 1) The Critical Phase, 2) The Visionary Phase, and 3) The Realistic Phase. | Children could articulate their thoughts, ideas, and visions for a better and healthier school environment. Identified problem areas and solutions differed widely and represented a broad perspective of health including social, physical, environmental, and emotional aspects. |
| Danielson et al., 2017 | To provide a perspective on health education practice, and how democratic health education is still not prioritised in the everyday school practice and implementation of the latest reform in Danish schools. | Interactionist, Ethnography | Qualitative: 1) Field notes, 2) Group interviews with students | Two classes from different public schools, both situated in the same large city in Denmark, participated across two school years. One of the schools had a diverse pupil composition in terms of cultural, ethnic, and socioeconomic backgrounds. The student composition in the second school was more homogeneous, and mainly consisted of students of Danish ancestry, from more socioeconomically well-off families. | Students (n = unknown) | 13-15  (Grade 7-8) | Health in Virtual Spaces: Aimed to generate reflection among the students about how their individual and collective perceptions and practices of health were influenced by the frequent health messages to which young people are exposed in their everyday virtual lives. | Conservative and neoliberal thoughts enforce school priorities and are barriers to more democratic school health practices. |
| deJongh et al., 2014 | To explore a group of learners’ experiences of their participation in a leadership camp that was part of a Health Promoting Schools (HPS) project and how this developed their leadership skills. | Explorative, Qualitative approach | Qualitative: 1) Focus groups with students | A cluster of 3 high schools in Cape Town. | Students: (n = 6) | 16 -17 years  (Grade 9 -11) | Activities included public speaking, and talent shows to build self-esteem, decision-making, communication skills, understanding gender, power and group dynamics, assertiveness, and team building and other fun activities. Reflective writing took place at the end of the day. | The opportunity for students from low-socioeconomic backgrounds to be part of a Health Promoting Schools (HPS) project in a safe and alternative manner can bring about deep and lasting personal changes in the learners. |
| Fletcher et al., 2015 | To explore the process of involving students and staff on school action groups, and staff and student experiences of reviewing local data and initiating school-level changes, to address bullying and other aggression. | Clustered randomised control pilot trial | Qualitative: 1) Interviews with school management team members, other action group members, and facilitators, 2) Focus groups with school staff and students, 3) Observations of action group meetings | Eight mixed-sex secondary schools in London and South-east England with allocation to either an intervention initiating change locally in bullying and aggression through the school environment (n = 4) or continuation of normal practice (n = 4). | School management teams: (n = 7)  Action group students: (n = 14)  Action group staff: (n = 9)  Student focus groups: (n = 112, Staff focus groups (n = 20) | Students: 12 -13 years  (Grade 8) | INCLUSIVE: An intervention that initiated change locally in bullying and aggression through the school environment. This intervention combined changes to the school environment with the promotion of social and emotional skills and restorative practices. | Main takeaways included that the establishment and sustainment of these groups from the intervention were impacted by traditional features of English secondary school, the involvement of external facilitators limited the potential for involvement of young people to lead, and ensuring a head teacher took part in an action group was important for the group to have power to enact change. |
| Gadin et al., 2009 | To analyse if young students could be substantive participants in a Health Promoting Schools (HPS) project. The specific aims were to analyze the changes the students proposed in their school environment, how these changes were prioritized by a school health committee and to discuss the students’ proposals and the changes from a health and gender perspective. | Participatory Action Research, Case study | Qualitative: 1) Document review | One elementary school with students in grades 1 - 6. | Students (n = approximately 150) | 6 - 12 years  (Grade 1 - 6) | It's your decision: An intervention that started with a 5-day program of information about health, gender theories and gender pedagogy for all teaching staff, to create a common knowledgebase and to introduce the teachers to the health education model called “It’s your decision” that was used in the project. | The analysis showed six categories of the students’ proposals: social climate, influence on schoolwork, structure and orderliness, security, physical environment, and food for well-being. Teachers’ priorities corresponded to the students’ categories but had an additional category regarding health education. |
| Garnett et al., 2019 | To outline and describe a developmentally appropriate curriculum (Getting to the Y) to engage youth as change agents in their school health community through youth-led research activities with publicly available and locally derived data from the Youth Risk Behavior Surveillance Survey. | Participatory Action Research | Qualitative: 1) Focus groups with students | Approximately 40% of middle and high schools in Vermont, United States participated in the intervention Getting to the Y (GTY) and the program has been replicated in both Native American and large urban schools in New Mexico since 2012. In total, 33 schools (2007-2012), 77 schools (42 new; 2013-2018); 15 schools with 100 students taking leadership roles (2017-2018). | Students (n = 17) | NR  (NR) | Getting to the Y (GTY): GTY was a Youth Participatory Action Research initiative, whereby students analyzed their own school health data and used those data to make real change in their school community. The initiative was organized around the four key processes of peer-to-peer training, data analysis, a community dialogue event, and action. | Results from the focus group reinforced the GTY principles and reinforced the importance of structured opportunities for youth. Results also indicated that GTY is a scalable, developmentally appropriate, resource-efficient, and an empirically based curriculum. |
| Griebler et al., 2012 | To summarise the effects of student participation in student councils, to show who benefits most, and to discuss characteristics that make student participation in student councils successful/ effective. | Document review | Qualitative: 1) Document review | The article is based on a recently conducted systematic literature review on the effects of school health promotion on students. Nearly one third of all included cases were dealing with student participation in the form of student councils. | NA | 5-19 years  (Primary and secondary grades) | NA | Effects of student participation in student councils were categorised into personal effects on students, effects on interactions and on the school as an organisation. Students who participated in councils, (i.e., the student representatives) personally benefited the most. All students benefited from improvements in the  physical or social environment of the school. Characteristics that made student  councils successful included the council composition, election procedures of representatives, positions  and procedures and frequency and timing of council meetings, communication between council and  the student body or other actors, the decision-making power of the council, supportive school context  and training of councillors and staff. |
| Gutuskey et al., 2016 | To examine how experiences as part of a healthy school leadership team impacted youth members. In particular, how do young people believe that leadership experiences influence their healthy eating, physical activity, and leadership development. | Empowerment theory, Case study, Grounded theory | Qualitative: 1) Interviews with adult advisors and students, 2) Observations | One suburban elementary school in the Midwestern, USA formed a student-led school health improvement team focused on healthy eating and physical activity. | Students (n = 9),  Adult advisors (n = 2) | 8-10 years  (Grades 3-4) | Student-led school health improvement team focused on healthy eating and physical activity. | Participants identified improved leadership skills and improved healthy eating and physical activity behaviours as outcomes from their emerging identities as health leaders. |
| Holmberg et al., 2018 | To describe adolescents’ experiences of participating in a health promoting school-based intervention regarding food and physical activity with a focus on empowering aspects. | NR | Qualitative: 1) Focus groups with students and teachers | The school was in an urban, disadvantaged community in Sweden. | Students: (n = 49), Teachers: (n = 4) | Students: 14 - 15 years (NR) | The three-semester long intervention had a focus on healthy food and physical activity habits. The specific intervention components were developed and implemented through shared decision-making between the researchers and the participants. The intention of the intervention was to enable empowerment through partnership with the adolescents by letting them express their perceived health related needs, discussing how to access health information and by facilitating skills development. | A theme was generated, intersecting with all the categories: Gaining control over one’s health. Participants appreciated influencing the components of the intervention and collaborating with peers in active learning. Participants also reported gaining new health information, found inspiration in new activities and the use of pedometers and photo-food diaries helped them reflection their health behaviours. The participants experiences were also echoed by their teachers. |
| Holsen et al., 2015 | To explore the experiences of peer leaders, teachers and principals who were part of the Dream School Program. | Participatory | Qualitative: 1) Focus groups with students and teachers, 2) Group interviews with principals | Three upper secondary and two lower secondary schools were included in the study. These  were five of the six schools which have implemented the Dream School Programme in  Norway. The schools had all used the programme for at least 1 year. | Students: (n = 25), Teachers: (n = 23), Principals: (n = 5) | Students: 14 - 19 years  Teachers: 30 - 60 years  Principals: NR  (Grades: Upper and lower secondary schools) | Dream School Programme: A universal school intervention that aimed at creating good psychosocial learning environments and positive mental health to strengthen students’ motivation to stay in school. The main foundations of the programme comprised of a health promotion perspective, participatory learning activities and peer-support initiatives as a vehicle for youth involvement and development. | Benefits to being a peer leader included positive feelings of helping others and feelings of responsibility; acquiring new skills, gaining new confidence and feelings that they made a difference. The teachers and principals reported that the peer leaders contributed to a more secure class environment. |
| Hutchinson et al., 2012 | To investigate strategies used by school-based nurses to promote adolescent boys access to and engagement with school-based health services. | Constructivism, Grounded theory | Qualitative: 1) Interviews with school nurses | Two rural schools, 14 metropolitan schools. | School-based health nurses: (n = 10) | 30-50 years  (NR) | School-based health services. | The main finding related to creating connections including three inter-related sub processes: facilitating communication, mediating contextual issues, and bridging cultural issues. |
| Jensen and Simovska, 2005 | To present and discuss two models of student participation, developed in the context of Health Promoting Schools (HPS) in two different cultures/countries (Denmark and Macedonia). | NA | NA | NA | NA | NA  (NA) | NA | NA |
| Jensen, Simovska, Larsen, Holm et al., 2005 | To build on Young Minds, an educational approach developed and carried out by a team from the Research Programme for Environmental and Health Education at the Danish University of Education. | Participatory Action Research, Case study | Qualitative: Different based on case study | The project group consisted of primary and secondary school students from eight classes representing the Czech Republic, Denmark, England, Finland, Hungary, Ireland, Slovenia and Spain as well as their respective teachers. In addition to this, a coordinating team of four researchers from the Research Programme for Environmental and Health Education at the Danish University of Education was responsible for overall project management and facilitation. | Varied by case study | Varied by case study (Grade: Varied by case study) | Young Minds: This initiative was an Internet-based project in which young people from eight countries in Europe explored links between youth, culture, health and environment. | The report concluded that student participation is one of the crucial constituents of the Young Minds educational approach. Student participation means the genuine involvement of students in processes relating to the content and processes of learning about health and the environment. |
| John-Akinola et al., 2014 | To facilitate a three-phase participatory research process to document the views of children about participation in school. | Participatory research | Qualitative: 1) Focus groups with students, 2) Reflective writing with students | Students from three primary schools in Ireland. | Students (n = 248) | 9 - 13 years  (Grade 4-6) | NA | The most common categories that made pupils feel a part of their school were school uniforms, sports, friends, teachers, and their school/classroom environment. Increase in the number of school activities, encouraging friendship and equal participation were key indicators of how pupils would ensure that everybody felt a part of the school. The findings also indicated that interpersonal relationships and belonging are important. |
| Joint Consortium for School Health Partnership and The Students Commission, 2018 | This Toolkit is intended to:  1) Help communicate the importance of youth engagement as a key approach to implementing comprehensive school health, 2) Provide research and rationale for practicing youth engagement in schools, school boards and districts, government ministries, health regions, and community organizations, 3) Provide a “how-to” resource of effective practices to support youth engagement in these contexts. | NA | NA | NA | NA | NA,  (NA) | NA | Effective youth engagement leads to positive outcomes for young people at three levels: 1) The Individual level: e.g., increased personal skills, healthy choices, and sense of identity, 2) The social level: e.g. stronger positive connections with friends and adults, and a larger support network, 3) The system level: e.g. greater civic engagement, policies and programs responsive to the needs of young people, and new creative ways to govern. |
| Kinman et al., 2017 | To utilize constructivist principles to strengthen a collaborative partnership between a pediatric residency training program and a classroom of at-risk high school students to improve adolescent health, while simultaneously developing a unique experiential learning activity that educates pediatric residents and at-risk high school students in social justice and the social determinants of health. | Constructivism, Participatory Action Research | Qualitative: 1) Reflections, 2) Observations, 3) Document review | Pediatric residents rotating on the Adolescent Medicine rotation and the high school students enrolled in the Women’s Alliance elective class at the participating high school. The high school population consisted of a total of 2,410 students and predominantly of minority students, with 72.6% of the student body that year considered Hispanic or Latino. Nearly 90% of the student body were considered socioeconomically disadvantaged. | Students: (n = approximately 25, 12 active),  Teachers: (n = unknown),  Pediatric residents: (n = unknown) | Students: NR  (Grade 9-12) | Participatory Action Research projects co-developed with pediatric residents and students. | Student reflections revealed evidence of educational transformation, critical use of technology, and personal growth, while resident reflections revealed improved communication skills with adolescents and increased knowledge of the social determinants of health that affect adolescents in their community. |
| Kontak et al., 2022 | To understand youth perspectives on Health Promoting Schools (HPS), as well as how they are engaged in school decision-making. | Pragmatism, Youth Participatory Action Research | Qualitative: 1) Interviews with students | Ten youth were trained as peer researchers. The peer researchers interviewed 23 of their peers on perspectives related to HPS and school youth engagement across schools in Nova Scotia, Canada. | Student researchers (n = 10),  Student interviewees (n = 23) | Student researchers:  12 - 16 years (grades 7 – 10)  Student interviewees: NR (Grades 7 – 10) | NA | Themes related to a healthy school community were mapped onto the pillars of HPS: 1) Social and Physical Environment, 2) Teaching and Learning, 3) Partnerships and Services and 4) School Policies. Participants placed more importance on the social and physical environment of the school including respect, inclusivity, supportive relationships and the design of spaces. Key factors for youth engagement were: 1) Safe and supportive spaces, 2) Passion and interest, 3) Using their voice, 4) Power dynamics, 5) Accessibility and 6) Awareness. |
| Lofton & Dewey-Bergren, 2019 | To provide an overview of using photovoice in school health promotion to collaborate with youth. | NA | NA | NA | NA | NA  (NA) | Photovoice is typically conducted in several stages: 1) Select and recruit community leaders and/or policy makers, 2) Recruitment of students, 3) Photovoice training, 4) Informed consent; 5) Discuss and designate topics for photo assignments, 6) Distribute cameras and instruct on using them, 7) Provide time to take photographs, 8) Meet to discuss photographs, identify themes, and begin data analysis; and 9) Reach out to policy makers and develop an action plan. | Photovoice is a community-based approach that is well suited for the school environment, encourages engagement from youth and staff and provides a platform for student voice. |
| Lopresti et al., 2021 | To describe the key characteristics of successful Indigenous Youth Mentorship Program delivery. | Participatory, Ethnography | Qualitative: 1) Field observation notes, 2) Focus groups with Indigenous youth mentorship program participants, 3) Semi-structured interviews with young adult health leaders and youth mentors. | 13 Indigenous school communities across Canada. | Focus groups: Young adult health leaders (n = 8), youth mentors (n= 8)  Follow up interviews: Young adult health leaders (n = 1), youth mentor (n = 1) | 13-18 years (NR) | Indigenous Youth Mentorship Program (IYMP): A peer-led health promotion program developed for elementary school students in Indigenous school communities in Canada. A local young adult health leader and high school youth mentors offer students healthy snacks, physical activity games, relationship building activities and cultural teachings. IYMP aims to improve children's health and wellbeing and empower Indigenous youth and communities. | Five key characteristics were identified as important for success in the Indigenous Youth Mentorship Programs delivery including: 1) Building relationships, 2) Communication, 3) Community engagement, 4) Instilling a sense of ownership, and 5) Program support. |
| Lyons et al., 2002 | To discuss the evolving role of pupils and staff in school and explore the implications this may have on their health education and for those responsible for school health policy. | NA | NA | School Councils UK developed out of successful community participation work carried out by Priority Area Development (PAD) in inner city areas in Liverpool, United Kingdom. School Councils UK runs training for primary, secondary and special schools. | NA | NR  (Primary, secondary, and special schools) | School Councils UK was an educational charity that trained teachers and children to help them set up structures that involve pupils in discussion and decision making about things that affect their lives. | Two key factors to achieve successful student councils included supportive staff and effective structures. |
| Mwanga et al., 2007 | To explore the idea of pupils as active dialogue partners in schools and as active change agents in the local community and the families in the Tanzanian context. | Action participation and Action competence, Cross-sectional | Qualitative: 1) Focus groups with students, teachers, and parents, 2) Interviews with students, teachers and parents | A cross-sectional study was carried out in a rural part of Magu district, Tanzania. Participants were recruited purposively among pupils and teachers from four primary schools and parents from three surrounding villages of Nyanguge area. Most of the study participants belonged to the Sukuma tribe (a Bantu-speaking people who live in part of Sukumaland, located to the west and south of the Lake Victoria. | Students: (n = 119),  Teachers: (n = 40),  Parents: (n = 147) | Students: 0 - 19 years (NR)  Teachers/Parents: 20 - 60+ years  (Primary School) | NA | Participants favoured an approach where school children played an active role as health change agents in a combined school and community health education project. This conclusion contradicts traditional views in many African cultures where power, status and wisdom are usually closely associated with old age. A number of barriers to student engagement were found, including the curriculum, time constraints, class size, teaching materials and teachers’ skills and working conditions. |
| Nakiwala et al., 2016 | To develop a better understanding of how children can transform from mere recipients to active partners in malaria control efforts, by exploring the implementation of a school health education program in Uganda. | Process evaluation, Multi-case study | Qualitative: 1) Focus groups with students, 2) Interviews with teachers and health promotion staff | The study was conducted in Masaka and Hoima, 2 districts, which were purposely selected having been the first to embrace the program. Masaka is located 120 kilometers south, while Hoima is 230 kilometers, Southwest of Kampala, capital of Uganda. At the time of the study in July 2013, the two districts had implemented the “Stop Malaria in Your Community” program for three and a half years. At the time of the study, these schools had an average of 350 children, both boys and girls, who were between six and 13 years of age. | Students: (n = 72) Teachers: (n = 6)  Health promotion staff: (n = 14) | Students: 10 - 13 years  (Grades 5 to 7) | “Stop Malaria in Your Community”: A program aimed to enhance capacity for malaria prevention and treatment in 34 districts. Two of the program components were the implementation of malaria control education and actual malaria prevention activities. | Children acted as health messengers, offered peer support and engaged in environmental management to minimize mosquito breeding. The benefits of the school malaria program included increasing access to malaria information, boosting malaria knowledge, improving children’s self-esteem and their skill as health educators. Barriers to implementation included hostility from adults, inadequate time and tight school schedules. |
| National Assembly on School Based Health Care, 2011 | To describe engaging youth in school-based health care. | NA | NA | NA | NA | NA  (NA) | School Based Health Centers (SBHC): Youth engagement can take on a variety of forms in the SBHC setting. Some exist solely to promote their own SBHC. Others broaden their activities to local, state, or even national advocacy for the SBHC. | Youth engagement can enhance the basic building blocks of SBHCs including community assessments, outreach and promotion, and service delivery. Youth can also support policy and advocacy efforts for SBHCs and the broader school health movement |
| Nilsson Lindström et al., 2022 | To examine middle school-aged pupils’ participation in an action-oriented school health promotion project and investigate its effects on their health experience, learning and influence. | Action-oriented research | Quantitative: 1) Survey to students (eight times) | Countryside school in southeast Sweden during 2018 and started with school representatives inviting researchers into the school to cooperate in a health promotion project. The school, from preschool to 9th grade, had 320 pupils and 30 employees. Only the middle school pupils (grades 4–6) and their teachers participated in the health promotion project. The project activities were conducted from February to May 2018. | Baseline students: (n = 85)  Follow-up students: (n = 73) | 10 - 13 years  (Grades 4 to 6). | The main purpose of the health promotion project was for the middle school to develop and implement a multidisciplinary health intervention to increase metacognition about health and learning among the pupils. The intervention meant that the teachers included health as a running theme when teaching language development, natural science, mathematics, aesthetics and physical education every week. | Health differences were found between gender and age. Aspects of pupil influence and learning about health showed positive progression during the project. Satisfaction with school performance was most associated with health experience. Thus, health experience, gender, and age are factors to consider when planning similar health promotion projects in schools. |
| Nykia et al., 2022 | To understand how immigrant students imagined, felt and thought about themselves in relation to education and health-related programmes from their perspective as Nova Scotia, Canada school stakeholders. | Critical race theory, Social constructivism, Participatory | Qualitative: 1) Photovoice with students, 2) Interviews with students, 3) Focus groups with students | Secondary school students of colour who had migrated to Nova Scotia, Canada from Africa and the Caribbean region within the last 10 years. | Students: (n = 15) | 12 - 21 years  (Secondary students) | Photovoice workshop: Initial face-to-face preliminary individual interview to establish each participant’s biographical circumstances. The study adopted the photovoice protocol of Warne et al. (2013) as a guide to data collection. It outlined three main stages: Introduction to photography, taking pictures and discussion of photos. | Three overarching themes were developed from the study: 1) Pedagogy of the Health Promoting Schools (HPS), 2) Black consciousness and 3) School health culture. |
| Orme et al., 2013 | To examine students’ role in the implementation of a whole-school food program by exploring: 1) The nature and extent of student participation; 2) Perspectives of school staff; 3) Perspectives of student participants; 4) Implications for policy, practice and research. | Case study | Mixed method: 1) Survey with teachers, 2) Follow-up questionnaire, 3) Interviews with staff, 4) Focus groups with students | Seventy-five primary schools participated in the Food for Life Partnership programme (open to primary, secondary, and special schools). Study participants were 77 students (year 3 and year 5) from six schools. | Students: (focus groups n = 77)  Teachers: (total n = unknown, interviews n = 24) | NR  (NR) | Food for Life Partnership Program: A multi-level initiative that used a whole-school approach to promote healthier nutrition and food sustainability awareness for students and their families. Schools focused on improvements in quality of school food provision, health education, participation, collaboration and structural and policy change. | Student action groups play a pivotal role in catalysing and embracing a whole school approach and must be seen as an important mechanism for any health promotion strategy in a complex school environment. |
| Physical & Health Education (PHE) Canada, 2023 | To provide educators and administrators with a new way of implementing school-based initiatives by engaging students in the planning, design and implementation process. | NA | NA | NA | NA | NA (NA) | A 9-step approach to student-centered learning for school-based initiatives  Step 1: Identify the problem,  Step 2: Pre-initiative survey or interviews  Step 3: Convene the action team,  Step 4: Knowledge transfer,  Step 5: Design workshop,  Step 6: Planning for implementation,  Step 7: Solution implementation,  Step 8: Post-initiative survey or interviews  Step 9: Evaluate and refine. | NA |
| Pridmore et al., 2000 | To present a rationale for children’s participation and the impact of determinants of health on their participation. It also reviews children's participation models and uses one of these models to analyse the level of participation illustrated in case studies of school health in Nepal, Zambia and Botswana. | NA | NA | Presentation of case studies from Nepal, Zambia, and Botswana. | NA | NA (NA) | NA | This paper argued that children’s capacity for participation is determined not only by age and by stage of development but also by such factors as gender, religion, ethnicity, wealth, and disability.  It also concluded that among the main barriers to children’s participation are the attitudes of adults who have yet to fully realise the value of children’s participation and to develop the skills needed to work with them as partners for health. |
| Ruge et al., 2016 | To examine how students’ participation in an integrated school food program was related to the development of components of food and health-related action competence (F & HRAC). | Action-oriented research, Case study with sub-cases | Qualitative: 1) Observations, 2) Interviews with students, 3) Video, 4) Document review | Three subcases of student’s involvement in LOMA at three different stages considered three sub-cases. | Students: (Case A, n= approximately 100), (Case B, n = 28), (Case C n = 36) | Case A: 13 - 14 years (Grade 7)  Case B: 13 - 14 years (Grade 7)  Case C: 14 - 15 years (Grade 9) | LOMA-Local Food: An integrated education and health program called LOMA-Local  Food (LOMA). Practice in the LOMA program was based on a whole-school approach and activities took the point of departure in a set of principles, which served to secure the application of a local, healthy and sustainable approach to cooking, learning and public food procurement. | Students who participated in LOMA educational activities became motivated for  developing a food F&HRAC, which included components such as knowledge, insight, motivation,  ownership, action experience, commitment, cooperation and critical thinking. Students developed practical skills related to food and health, when they were cooking healthy school food together with  professionals and peers. The study also pointed to the importance of capacity building among teachers. |
| Simovska & Carlsson 2012 | To discuss the outcomes of the Shape Up intervention and challenges to students’ participation in school health promotion. | Multiple case studies | Qualitative: 1) Document review 2) Observations, 3) Interviews with local coordinators and teachers, 4) Group interviews with students | Shape Up intervention in 19 European cities (73 schools, 2300 students, 140 teachers). Study participation from 5 schools (Austria, Denmark, Italy, the Netherlands, and Spain). | Students: (n = 30),  Local Coordinators: (n = 10),  Teachers: (n = 10) | Students: 12 - 16 years  (NR) | Shape Up: A school-community approach to influencing determinants of healthy and balanced growing up. The project’s aims were consistent with the principles of critical health-promotion and health education theory and included involving pupils in influencing proximal determinants of healthy eating and physical activity. | Themes included: 1) More varied and enhanced provision for healthy eating and/or physical activity at school. 2) New or improved school policy concerning food and/or physical activity. 3) Improvements of the school physical environment. 4)Changes in the school’s local environment and new community partnerships. |
| Simovska et al., 2007 | To explore student participation through discussing a qualitative study, demonstrating a case for involving students actively in learning about health matters. Particular emphasis will be placed on teachers’ and students’ reflections relating to the process of participation in learning about health. | Constructivist; Interpretive; Case study | Qualitative: 1) Document review, 2) Interviews with students and teachers | The present case study is limited to 1) The first project phase as a whole (Young Minds 1) and 2) the project work of a few selected classes from the second project phase (Young Minds 2). A total of eight classes from eight countries (Denmark, the Czech Republic, Macedonia, Sweden, Iceland, Macedonia, Portugal and Slovenia) took part in the research. | Students: (n = 200, interviews, n = 8)  Teachers: (interviews, n = 8)  Facilitators (n = 8) | Students: 13 - 16 years  (NR) | Young Minds: An educational development project that explored links between youth, culture, and health. Young Minds is an international project which students from a number of schools in different European countries collaborate on issues related to health. The purpose of the project was to generate new, action research-based knowledge on effective methods for engaging primary and early secondary school students in learning about health in an action and collaboration focused way. | The analysis of the case study illuminated the trajectories of participation in which students learned about health in intentional, relational, and purposeful ways. These participation trajectories were viewed as situated in activity structures consisting of a variety of mutual interactions and different forms of participation. |
| Simovska et al., 2004 | To address the issue of student participation from the perspective of the health promoting schools' initiative by distinguishing between two different qualities of participation: genuine and token participation, as well as discussing a process evaluation related to a health promoting schools project in Macedonia. | Participatory, Process evaluation | Qualitative: 1) Evaluation workshop, 2) Structured interviews with teachers | Three schools with approximately 300 students and 30 teachers participated in the program. The programme took place over the course of nine months. Teachers and school staff were involved in an evaluation workshop to develop indicators for the process. | Teachers: (n = 15) | NR(NR) | Virtual Classroom - ICT, Changes and Learning: One of the recent programs within Health Promoting Schools (HPS) in Macedonia. It was the first Internet-based collaborative project since the national network was established. In addition to providing schools with equipment and teaching resources, the programs overall aim was to explore the possibilities and barriers connected with the use of new technologies (ICT) in participatory, action-oriented health education and health promotion. | One of the key elements of a HPS is appropriate `space' for the students to participate genuinely in relevant aspects of decision-making processes at school. A participatory approach to health promotion implies more than the improvement of the health status of individuals in a given school community. |
| Simovska et al., 2004 | To address the issue of student participation from the perspective of the Health Promoting Schools (HPS) initiative. It draws on experience from the Macedonian Network of Health-Promoting Schools and its collaboration with the Danish as well as other country networks within the European Network of Health-Promoting Schools. | NA | NA | Macedonia joined the European Network of HPS in 1995 with 10 elementary schools. Since then, the national network of HPS (Macedonian Network of HPS) has been established and structured in three rounds of schools, of which 34 are elementary and six secondaries. | NA | NA  (Primary and Secondary) | NA | Schools are considered ideal educational social institutions that are in the position to endorse these values, and educate young people to be competent, creative and responsible participants in such a society. |
| Society for Public Health Education (SOPHE), 2020 | To explain why youth voice is important to the Whole School, Whole Community, Whole Child (WSCC) model and how implementing youth voice can help healthy schools. | NA | NA | NA | NA | NA  (NA) | Whole School, Whole Community, Whole Child Model: A unified and collaborative approach to learning and health. | Engaging youth in designing, planning, implementing, and evaluating school health can help them become passionate about health and wellness and model healthy behaviors for other students. Youth voice can provide a pathway for students to contribute to school culture and provide a sense of safety, belonging, and self-efficacy. Youth voice can increase involvement in social and policy issues, strengthen community connections, and improve achievement. |
| Soleimanpour et al., 2008 | To describe two case studies and lessons learned in the implementation of youth-led community-based participatory research aimed at improving school-based health centers (SBHC) programs and policies. | Community-based participatory research, Case study | Case Study 1: Quantitative: Survey of students  Case Study 2: Quantitative survey of students | Alameda County School-Based Health Center Coalition (SBHC Coalition), a group of middle and high school health centers providing comprehensive health services to students. | Student Research Teams: (n = 2 to 6 students per team)  Case Study # 1: Students (n = 359),  Case Study # 2: Students (n = 260) | Student Research Teams: NR (Grades 8 -11)  Case Study # 1 & 2: NR (NR) | School-based Health Services Centres (SBHC) help develop and implement school health policies. Providing opportunities and training that enabled youth to identify and research the health needs of their peers, as well as advocate for improvements in SBHCs based on their research findings, represents an exciting youth development strategy. | Youth voice can play a crucial role in advancing policies that are of mutual concern and, at times, can expedite policy and program efforts that have been stymied. SBHCs can clearly benefit from youth perspectives on how to better address health issues in their school communities and make policy and programmatic improvements based on their research and recommendations. |
| Sonn et al., 2011 | To describe how learners from three secondary schools in poor, suburban, so-called coloured communities on the Cape Flats in the Western Cape, South Africa, participated in a Participatory Action Research project, referred to as Learner Voice. | Participatory Action Research | Qualitative: 1) Reflective writing with students, 2) Photovoice with students, 3) Interviews with students | Ten learners from three school were invited to volunteer to participate in the project. All the learners were involved in the Health Promoting Schools (HPS) project at their school. A few were prefects and/or members of the Representative Council of Learners. | Students: (n = 30, n = 18 core group who participated in all activities) | Students: 13 - 18 years  (NR) | Learners Voice: The Health Promoting Schools (HPS) project, started in 2008, was an on-going project in three secondary schools with volunteer teachers and learners and an interdisciplinary group of researchers at the University of the Western Cape (UWC). The Learner Voice research study aimed to give learners a voice, challenge learners and their thinking through creative exercises and experiential learning activities, stimulate learners to improve their schools as HPSs. | Children’s participation in research should be more than their token inclusion and the use of direct quotes as representative of “voice”. Acknowledgement of the imbalances of power, status and expertise in the research relationship and the necessity of building relationships of trust and mutual respect should be given as well as ways in which these imbalances and relationships were worked with. |
| Stjernqvist et al., 2018 | To examine the effect of the Health Promoting Schools (HPS) intervention ‘We Act–Together for Health' on children’s cognitive social capital. | Quasi-experimental, Controlled pre- and post-intervention study with a three-level cluster design, Process evaluation | Mixed methods: 1) Field visits, 2) Interviews (pre- and post-) with students, 3) Questionnaire with students | The was sample nested within 8 schools and 30 classes. The schools were in both suburban and rural settings and varied in size (ranging from approximately 300 to 1200 pupils). The schools varied with respect to the children’s socio-economic background. | Students: (n = 656) | 10-12 years  (Grade 5-6). | We Act–Together for Health: A HPS initiative with three components (school, health education and a parental component), all grounded on a broad and positive concept of health. We Act-Together for Health built on the IVAC (Investigation, Vision, Action, Change) methodology. | Child participation in health education can affect the children’s sense of belonging in the school, though without sufficient management support, may have a negative effect. |
| Sulz et al., 2016 | To gain insight into the experiences and motivations of teachers and students involved in a choice-based Comprehensive School Health model – Health Promoting Secondary Schools (HPSS). | Multisite randomised control trial, Community-based research | Qualitative: 1) Document review, 2) Focus groups with students and teachers | Participants included Action Team Members and Physical Education and Health Education teachers from the five HPSS intervention schools. | Action Team: (total n = 34, students n = 26, teachers n = 8)  Physical/Health Education Teachers: (n = 23) | Students: NR  (Grades 9 -12)  Teachers: NR (Grade 10). | Health Promoting Secondary Schools (HPSS): A Comprehensive School Health and self-determination theory (SDT) inspired intervention designed to improve the physical activity and eating behaviours of Grade 10 students in five intervention schools and five wait-list control high schools. | Analysis of the data revealed five themes associated with participants’ experiences and motivational processes:  1) Lack of time for planning and preparation, 2) Resources, workshops, and collaboration, 3) Teacher control impacts of student engagement, 4) Teacher job action inhibited implementation of HPSS action plans and, 5) Choice-based design impacts participants’ experiences. |
| Tomokawa et al., 2020 | To conduct a document review on the important, recent trends, and enabling factors of child participation in school health in Japan. | NA | Synthesis: Literature review | Elementary to high schools in Japan. | NR | NR  (NR) | NA | Four main findings were outlined including: 1) Having an explicit legal basis for participatory activities at the national level, 2) having clear notification, in relevant administrative documents at the prefectural and municipal levels, of the necessity for children’s voluntary participation, 3) Establishing a system for teachers to provide support for participatory activities at the school level and 4) Having a shared understanding among stakeholders about the pedagogical importance of participatory activities in school health. |
| UpLift Partnership, 2022 | To describe several aspects of the UpLift partnership work including school engagement and action as well as students and youth engagement. | Process and outcome evaluation | Mixed-Methods: 1) Focus groups 2) Interviews 3) Story sharing, 4) Tracking forms for adult champions 5) Health Promoting Schools Assessment Tools | 5 school regions across Nova Scotia, Canada. | Students (n = 215), Staff Champions (n = 18), Youth Engagement Coordinators (n = NR) | NR (NR) | The UpLift Partnership: A school-community-university partnership supporting the health and learning of school-aged children and youth using a Health Promoting Schools approach. Specific to youth engagement, students received funding to design and lead a project that enhanced health or wellbeing at their school with the support of adults including Youth Engagement Coordinators and HPS leads. | Particular to youth engagement in HPS, Youth Engagement Coordinators and Health Promoting School Leads play a pivotal role in prioritizing and enhancing student and youth engagement. The YECs have helped to strengthen students’ voices in HPS work and build student leaders for HPS within schools. |
| Vanner et al., 2014 | To present the results of a qualitative study that examined the nature and extent of children's participation in the Save the Children's primary school Health and Nutrition (SHN) project in El Salvador. | Participatory, Case study | Qualitative: 1) Field visits, 2) Interviews with students, 3) Focus groups with students, 4) Skits | As of 2011, the SHN was implemented in 44 rural primary schools in the districts of Sonsonate, La Paz and Ahuachapan (approximately 6500 pupils). Given the qualitative nature of the research, three out of the 44 schools were selected as sites. Student brigadiers from each school were elected by their classmates via secret ballot. | NR | Students: NR  (NR) | The School Health and Nutrition Project (SHN) project aimed to combine the enhancement of children's educational experience with heightened understanding of issues related to health and nutrition. | Children who were designated as School Health and Nutrition (SHN) project leaders (the student brigadiers) assumed substantial responsibility for project decision-making and appeared to have had a beneficial impact on school environments and among the student brigadiers themselves. |
| Warne et al., 2013 | To explore challenges and opportunities for applying photovoice in a school setting to support genuine participation. | Participatory Action Research | Qualitative: 1) Photovoice with students, 2) Interviews with students, 3) Focus Groups with students and teachers, 4) Document Review, 5) Reflective meetings with teachers | Two separate high school samples in O ̈stersund municipality, a medium-sized town in the northern part of Sweden.  Sample 1: Child Care and Recreation Program (CRP) group and Sample 2: Individual Program (IP). | Sample 1: Students (total n = 49, n = 7 research group 1, n = 7 research group 2, n = 35 teacher group)  Teachers: (n = 2) | Sample 1: Students: 18 - 20 years (NR)  Teachers: NR  Sample 2: Students: 16 - 19 years  Teachers: NR  (NR) | Photovoice was tested in 2 separate samples: Child Care and Recreation Program (CRP) and Individual Program (IP). The IP was designed for pupils with special educational needs, including reading and writing difficulties, neuropsychiatric diagnoses, and physical disabilities. | Photovoice challenges schools and society to have a better structure for genuine participation if youth participation is seen as valuable. Application of the photovoice method within a school setting has the potential to help schools achieve health-promoting environments more effectively. |
| Warren et al., 2019 | To evaluate fidelity, feasibility, and acceptability of action groups as part of a trial of a whole-school intervention to reduce bullying and aggression and promote health in English secondary schools. | Two-arm repeat cross-sectional randomized control trial | Mixed Methods: 1) Interviews with staff, students, external facilitators 2) Focus groups with staff and students, 3) Observations, 4) Survey to staff and students, 5) Document review, 6) Field notes | Learning Together intervention was implemented in 40 secondary schools across south-east England. The study population consisted of all students in the school at the end of Year 7. Our study population consisted of all students in the school at the end of Year 7 (aged 11–12 years) at baseline in March–July 2014, and at 24-month (end of Year 9; aged 13–14 years) and 36-month (end of Year 10; aged 14–15 years) follow-up. | Students: (n = varied based on data collection measure)  School staff: (n = varied based on data collection measure)  External facilitators: (n = varied per school) | Students: Baseline: 11-12 years  24 months follow-up: 13 - 14 years  36 month follow up: 14 - 15 years.  (Grades 7 – 10). | INCLUSIVE: Learning Together Intervention included action groups and drew on data on student needs. The intervention aimed to coordinate implementation of restorative practices and a social and emotional competencies curriculum; review policies and rules; and enact local decisions to modify school environments. | Action groups are a promising strategy for leading whole-school health promotion. Implementation is supported by external facilitation, local data and involvement of senior managers. |
| Wexler et al., 2017 | To describe process and outcome evaluation findings from the Youth Leaders Program (YLP) program during the 2013–2014 school year. | Process and outcome evaluation | Mixed Methods: 1) Focus groups with students, 2) Interviews with staff, 3) Surveys (pre- and post- school year) with students, 3) Document review (pre- and post-school year) | School district has approximately 2000 student's grades K-12, with schools ranging in size from 30 to approximately 650 students. Villages are linked by small airplane, boat, and snowmobile during the winter months, across a remote region encompassing 38,000 square miles. Each school in the district selects between four and 18 Youth Leaders. | Students: (total n = 764, survey n = 100 with 61 matched pairs)  School staff: (n = 13) | NR  (Elementary students: Grades 3-5, Middle school students: Grades 6-8, High School students: Grades 9-12). | Youth Leaders Program (YLP): A health intervention implemented in a rural Alaskan school district, which utilizes natural helpers and peer leaders to increase protective factors such as school engagement and personal/cultural identities, and to reduce risks associated with drug/alcohol abuse, violence, and bullying. | The Youth leadership Program improved school climate and increase school and other protective factors for participating students. |
| *NA = Not Applicable  ^NR = Not Reported | | | | | | | | |
