# Supplementary material for: The process of student engagement in school health promotion: a scoping review
Source: BMC Public Health. 2025 Mar 19;25:1063. doi: 10.1186/s12889-025-22121-8 (PMC11921550; doi:10.1186/s12889-025-22121-8)
Supplement: Supplementary file 6 — Supplementary Material 6 [file 12889_2025_22121_MOESM6_ESM.docx]

**Additional File 6. Reasoning for ‘Form of Engagement’ categorization by article**

| **Source Author** | **Level of Engagement** | **Reasoning (Form Explained)** |
| --- | --- | --- |
| Abildsnes et al., 2015 | 2 - Adult suggest, no dialogue, students accept or reject | Students choose between two pre-determined (teacher suggested) physical education models of "sports enjoyment" or "motion enjoyment." |
| Beattie et al., 2020 | 3 - Adult suggests, common dialogue, common decisions | Students analyze health data and decide on community change and action based on their perspectives, but initiative was led by an adult coordinator. |
| Boberova et al., 2017 | 5 - Students suggest, common dialogue, common decisions | Students were supported by an adult researcher to investigate different health issues that affected them, create visions about desirable changes, and act towards desirable change within their schools. |
| Bruselius-Jensen et al., 2014 | 3- Adult suggests, common dialogue, common decisions | Students were engaged in data collection and interpretation of an adult-led research project. |
| Clausen et al., 2019 | 5 -Students suggest, common dialogue, common decisions | Students took part in the future workshop, designed as a participatory forum for expressing concern and dissatisfaction with everyday life issues as well as ideas and visions for better living. |
| Danielson et al., 2017 | 3- Adult suggests, common dialogue, common decisions | Program and health messaging developed for students, students then were involved in data collection, analysis, and presentation of health messaging. |
| DeJongh et al., 2014 | 2 - Adult suggest, no dialogue, students accept or reject | Students participated in an adult-led and designed camp to develop leadership skills to later apply to Health Promoting Schools work at their school. |
| Fletcher et al., 2015 | 3- Adult suggests, common dialogue, common decisions | Formation of a school action group involving students and staff (supported by an external adult facilitator) to review, determine priorities, and develop and implement an action plan for changing the school environment. |
| Gadin et al., 2009 | 5- Students suggest, common dialogue, common decisions | Students were provided the ability to reflect and make suggestion changes for Health Promoting Schools, that were then implemented through a collaborative committee with adults. |
| Garnett et al., 2019 | 4 - Students suggest, student dialogue, students’ decisions | Participating adult-youth school teams attended a training day with peer-to-peer training and modeling of youth–adult partnership through co-facilitation. After the training, school teams continued to meet to plan and implement data analysis that is led by the students and supported by adult advisors. Students then organized and led a community dialogue event about their findings, with support from adults. Students took the lead on identifying community assets, reviewing results of their data analysis, refining an initial action plan, and identifying resources in the community that can be mobilized in support of change efforts. |
| Gutuskey et al., 2016 | 5 - Students suggest, common dialogue, common decisions | Student leaders were tasked with creating a healthier environment for their school alongside adult advisors. |
| Holmberg et al., 2018 | 3 - Adult suggests, common dialogue, common decisions | Health promoting school's activities were partly guided by participants expressed needs and then carried out with classroom teachers and adult researchers. |
| Holsen et al., 2015 | 2 - Adult suggest, no dialogue, students accept or reject | Health promotion programme involved participatory learning activities and peer support initiatives; students and teachers are both active participants in this established programme. |
| Jensen et al., 2005 | 5 - Students suggest, common dialogue, common decisions | Students, following the IVAC approach (Investigations, Visions, Action, Change), investigated several different issues and perspectives, created visions about better alternatives for the future and took action to bring about changes and come closer to these visions Health Promoting Schools. |
| John-Akinola et al., 2014 | 4 - Students suggest, student dialogue, students’ decisions | Students were involved in research through a three-phase participatory design: students generated data by responding to two questions from adult researchers, a second group categorised the data, and a third group analysed the data by creating schema to present their views. |
| Kinman et al., 2017 | 4 - Students suggest, student dialogue, students’ decisions | Students were paired with a pediatric residency program to develop peer-led teen health education programs. Students identified the health topic to be addressed, researched and discussed the implications of this topic on adolescent health, created surveys to assess their fellow students’ attitudes, beliefs, and personal experiences of the topic being addressed, analyzed the results of the survey, decided upon presentation plans, and designed, created, and implemented educational campaigns to raise awareness of this topic on campus. |
| Kontak et al., 2022 | 3 - Adult suggests, common dialogue, common decisions | Students were involved in developing interview questions and conducting interviews for a research project, but not involved in adult-led data analysis or interpretation. |
| Lopresti et al., 2021 | 3- Adult suggests, common dialogue, common decisions | Program is developed by adults and delivered by high school students to elementary school students through a mentorship program with a young adult health leader |
| Nakiwala et al., 2016 | 2 - Adult suggest, no dialogue, students accept or reject | Each school nominated one adult teacher as the "health master" who set up an after-school health club. At the health club, students were trained in public speaking, basic malaria prevention and presentation techniques. They were then asked to deliver malaria messages within and outside their schools using drama, malaria talks, songs, and poems, guided by the teachers. |
| Nilsson Lindstrom et al., 2022 | 3- Adult suggests, common dialogue, common decisions | A support group that included adults and students was developed and met twice a term during the project to discuss project implementation and the progress of metacognition about health and learning in relation to different project activities. There was also a project group of students and adult who met every 6 weeks to discuss the implementation of activities at class level and changes throughout the project. |
| Nykia et al., 2022 | 4 - Students suggest, student dialogue, students’ decisions | Students used disposable cameras to take pictures in relation to their perceptions of Health Promoting Schools followed by a photovoice interview with the adult researcher to discuss the photos taken. |
| Orme et al., 2013 | 3 - Adult suggests, common dialogue, common decisions | Initiatives were pre-determined by adults and involved students in cooking, growing, school consultations, and farm visits. |
| Ruge et al., 2016 | 3- Adult suggests, common dialogue, common decisions | Students participated in a large information meeting, the development of a new LOMA-local food logo, or a combined “Health and Math” project. The project focused was pre-determined by adults, but common dialogue with students for development and implementation. |
| Simovsaka & Carlsson, 2012 | 5 - Students suggest, common dialogue, common decisions | Source describes student participation in a Health Promoting Schools project at several schools; students were involved in activities that were determined by adults (sometimes in consultation with students), close guidance by teachers of students as they select and prioritise actions, or students as planners and organizers of activities. |
| Simovska et al., 2004 a | 5 - Students suggest, common dialogue, common decisions | Students participated in Young Minds, an international Web-based project in which students from a number of schools in different European countries collaborate on issues related to health. The IVAC (Investigation– Vision–Action–Change) participatory model was adopted to structure the student participation. |
| Simovska et al., 2004 b | 5 - Students suggest, common dialogue, common decisions | Student groups investigated the issue of student participation from different perspectives, comparing the situation in their own schools with other schools nationally and internationally. In addition, they shared information and ideas among themselves and created a Web page to communicate their findings. Students then school agreed upon and took actions to improve the opportunities for their participation in school decision-making processes. |
| Soleimanpour et al., 2008 | 4 - Students suggest, student dialogue, students’ decisions | Students were involved as student researchers, supported by an adult researcher, and implemented each step of their research project, including topic selection, instrument development, data collection, data analyses, and presentation of findings. |
| Sonn et al., 2011 | 3 - Adult suggests, common dialogue, common decisions | The schools are involved in a Health Promoting Schools (HPS) project and learners and teachers are engaged in various activities of the HPS project, such as learner camps, recycling clubs, peace clubs, TB-and HIV-awareness activities and related interschool health promoting activities. These projects are predetermined but involve common dialogue and participator approach with students. |
| Stjernqvist et al., 2018 | 5 - Students suggest, common dialogue, common decisions | Students collected individual and class-level data on healthy behaviours and students interpreted their data with teacher support. Next, students imagined their visions for Health Promoting Schools and then worked in teacher-formed groups to present their visions to students and adults. Then students, supported by adult educators, formed a health committee to take actions towards their visions. |
| Sulz et al., 2016 | 3 - Adult suggests, common dialogue, common decisions | Schools developed Action Teams (adults and students) who were provided funding to purchase equipment or services to support whole-school events and then they implemented whole-school events and policies to promote health. |
| UpLift Partnership, 2022 | 5 - Students suggest, common dialogue, common decisions | Students lead HPS projects from determining priorities to implementation with the support of youth engagement coordinators and other school health promotion staff. |
| Vanner et al., 2014 | 2 - Adult suggest, no dialogue, students accept or reject | Elected to their positions as student leaders in the project, the brigadiers are responsible for disseminating knowledge about good health and nutrition and for motivating their fellow students to contribute to health- related improvements in their respective schools, as prescribed by adults. |
| Warne et al., 2013 | 5- Students suggest, common dialogue, common decisions | Using photovoice, students took pictures of what made them feel and work well in school, participated in workshops to discuss photographs, and face-to-face interviews to understand students' experiences using photo voice. A survey was then distributed to adult policymakers to determine if the student's suggestions led or will lead to any changes in the organization |
| Warren et al., 2019 | 3- Adult suggests, common dialogue, common decisions | Schools implemented Action Groups of staff and students to coordinate intervention delivery and revise school rules, and enact local decisions informed by survey data to support restorative approaches to school discipline and make the school environment healthier. |
| Wexler et al., 2017 | 4 - Students suggest, student dialogue, students’ decisions | Student leaders organized a variety of recreational activities for students and their families, including making morning announcements, organizing community events, making classroom presentations, speaking at assemblies, helping during community meetings, performing needed tasks in the school, conducting behavioral interventions with younger students who misbehave, and reaching out if they see a student in need. |
